# Aggregation processes in customer rating systems - Insights from an economic decision experiment

**DOI:** 10.1371/journal.pone.0343851

**Published:** 2026-07-14

**Authors:** Dirk van Straaten, Behnud Mir Djawadi, Vitalik Melnikov, Eyke Hüllermeier, René Fahr

**Affiliations:** 1 Department of Management, Paderborn University, Heinz Nixdorf Institute, Paderborn, Germany; 2 Department of Computer Science, Paderborn University, Heinz Nixdorf Institute, Paderborn, Germany; 3 Department of Computer Science, Ludwig-Maximilians-University Munich, Institute of Informatics, Munich, Germany; 4 Munich Center for Machine Learning (MCML), Munich, Germany; 5 IZA@LISER Network, Luxembourg Institute of Socio-Economic Research (LISER), Esch-sur-Alzette/Belval, Luxembourg; Iowa State University, UNITED STATES OF AMERICA

## Abstract

The aggregation of rating metrics in reputation systems is crucial for mitigating information overload by condensing customer rating distributions into singular valence scores. While platforms typically employ technical aggregation functions, such as the arithmetic mean to capture product quality, it remains unclear whether these functions align with customers’ innate aggregation patterns. To address this knowledge gap, we designed a controlled economic decision experiment to elicit customers’ aggregation principles by analyzing their product ranking decisions and contrasting these with various reference functions. Our findings indicate that the majority of customers aggregate rating information in accordance with the arithmetic mean. However, a granular analysis at the individual level reveals significant heterogeneity in aggregation behavior, with a substantial cluster exhibiting binary patterns that focus equally on negative (1–2 star) and positive (4–5 star) ratings. Additional clusters concentrate on negative feedback, particularly 1-star ratings or 1–2 star ratings collectively. Notably, these inherent aggregation patterns exhibit stability across variations in numerical information presentation and are largely not significantly influenced by individual characteristics, such as online shopping experience or demographics. The only exception is a plausible relationship between risk attitudes and aggregation functions that focus on extreme ratings, such as 1-star, 1–2 star, and 5-star ratings. Our findings suggest that while the arithmetic mean captures the behavior of the majority of consumers, platforms could benefit from offering customizable aggregation options to better cater to diverse user preferences for processing rating distributions. By doing so, platforms can enhance the effectiveness of their reputation systems and improve the overall quality of decision-making for consumers.

## Introduction

Online markets provide consumers with a plethora of information sources to inform their purchase decisions, with customer feedback emerging as a particularly vital component. As the sole source of information not provided by manufacturers or selling platforms, customer feedback has become an indispensable resource for online shoppers. Indeed, research has shown that a significant majority of consumers, with over 80% consulting reviews during their purchase decisions [1], rely heavily on customer feedback to guide their purchasing behavior. Given the impracticality of manually processing large volumes of reviews, online platforms typically employ aggregation mechanisms to distill numerical ratings into single summary metrics representing the valence (i.e., the quality) of the underlying product. Prominent examples of such aggregation systems include Amazon’s 5-star rating system and Booking.com’s 10-point scale, both of which are designed to provide a concise, yet informative, representation of product quality through a single valence score.

The dominant approach to calculating valence scores is the arithmetic mean, which assigns weights to rating categories in proportion to their scale values. Although research has established a positive correlation between average ratings and sales performance [2], suggesting that average ratings are a useful indicator for consumers’ purchase decisions, a growing body of evidence suggests that consumers may process rating distributions in a manner that systematically deviates from arithmetic averaging. For instance, [3] found that average ratings often fail to accurately reflect objective quality measures, implying that consumers may employ alternative aggregation strategies that differ from the arithmetic mean in their decision-making process. Furthermore, the arithmetic mean’s susceptibility to outliers and strategic manipulation, as highlighted by [4], combined with evidence of systematic biases in online reputation systems [5,6], may lead consumers to rely on alternative aggregation methods. Moreover, recent studies have shown that consumer preferences for products can shift significantly in response to different rating distribution displays, as documented by [7] and [8], which further suggests that the arithmetic mean may not always be the primary method of aggregating distribution information. This accumulating evidence underscores the need to reexamine the assumption that consumers uniformly rely on the arithmetic mean to process rating distributions, and instead, consider the possibility that consumers may employ more nuanced and context-dependent aggregation strategies.

Despite the extensive theoretical discussions in the literature on properties of alternative aggregation functions, including informativeness, robustness, and strategy-proofness (see [4,9] for comprehensive reviews), we lack empirical evidence on how consumers actually aggregate rating information in practice. Elucidating these natural aggregation patterns is crucial for market design, as it has important implications for three key aspects: first, it reveals the information cues that drive consumers’ quality assessments; second, it enables platforms to evaluate the effectiveness of the arithmetic mean as decision aid for consumers; and third, it may suggest alternative aggregation functions that better align with actual consumer decision processes, potentially leading to more informed and effective decision-making.

This paper addresses this knowledge gap by employing a controlled laboratory experiment to investigate how consumers aggregate rating distributions in online markets. Specifically, we design an experimental framework in which subjects are presented with triplets of products from diverse categories (e.g., USB memory sticks) along with their corresponding rating distributions. We then ask subjects to rank these products in accordance with their individual preferences. In contrast to prior research based on hypothetical purchase intentions (e.g., [7]), our design elicits truthful preferences through an incentive-compatible mechanism where subjects face a higher probability of actually receiving their more preferred products, thereby ensuring that their ranking decisions reflect their genuine preferences. We analyze these ranking decisions using a Maximum-Likelihood estimation framework, which enables us to identify the aggregation function that best predicts consumer preferences and to test the proposition that the arithmetic mean accurately captures how consumers process rating information in online markets. By doing so, we aim to provide a more nuanced understanding of consumer behavior in online markets and to shed light on the effectiveness of the arithmetic mean as a decision aid for consumers.

## Aggregation processes in reputation systems

### Aggregation metrics background

**Fig 1:**
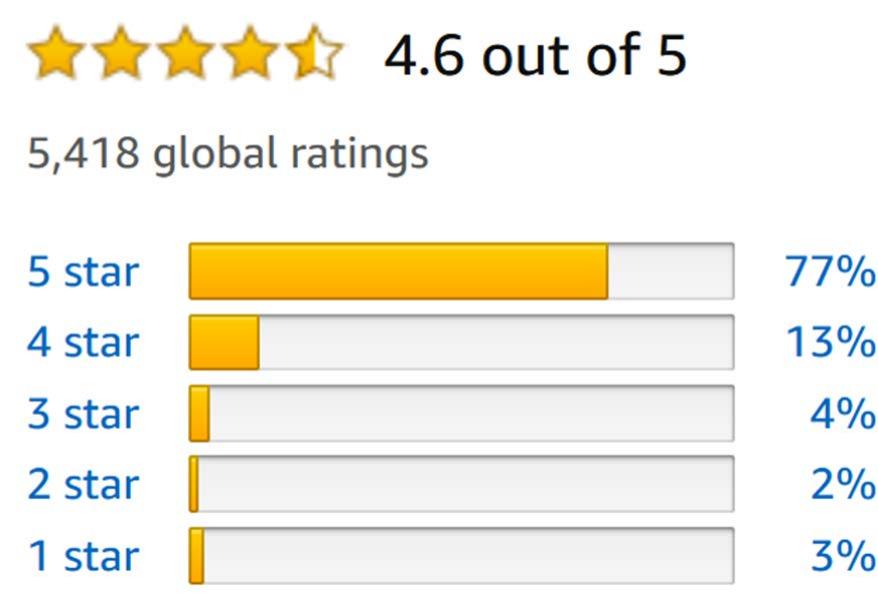
Example of customer rating distribution and calculated valence from an Amazon.com website.

Rating aggregation metrics synthesize customer feedback into concise parameters that summarize user evaluations (cf. Fig 1). The main purpose of aggregation metrics is to achieve comparability between products on marketplaces and to evaluate product quality.

According to [10], the digitization of word-of-mouth poses significant challenges, underscoring the importance of aggregating customer reviews to provide accurate assessments of sellers and products. Notably, [11] demonstrates that when customer reviews are presented in a disaggregated manner, outlier ratings are often discounted by consumers, who tend to attribute extreme evaluations to reviewer characteristics rather than product quality. Conversely, when reviews are aggregated and the distribution of ratings is displayed without revealing individual ratings, this effect is mitigated, suggesting that aggregated reviews can provide a more reliable indicator of product quality.

The work of [12] highlights the importance of visualization and summary statistics in facilitating accurate judgments, as it reveals that individuals in a hypothetical setting tend to systematically underestimate the occurrence of rare events when presented with a limited sample of individual ratings rather than complete distributions. This incomplete information can lead to suboptimal decision-making, as consumers often select products with lower mean ratings. Notably, the relationship between individual customer reviews and aggregation metrics is characterized by bidirectionality. Specifically, the overall rating of a product is not solely a function of individual assessments, but rather, the aggregate score itself exerts an influence on how users perceive the credibility of individual reviews that diverge from the collective evaluation [13]. This interdependence underscores the complex dynamics at play in the formation of consumer opinions and the critical role of aggregation metrics in shaping these opinions.

In addition to the *valence* of reviews, which refers to the perceived quality or goodness of a product, the number of ratings (*volume*) and the *variance* of the ratings also exert a significant influence. Research by [14] demonstrates that both valence and volume have a positive impact on sales elasticity, suggesting that higher-rated products with a larger number of reviews are more likely to experience increased sales. In contrast, [15] find that variance in customer ratings, which can be attributed to quality differences, has a negative effect on demand. However, it is essential to note that variance or bimodality in customer rating distributions are not necessarily detrimental. In fact, [16] show that, in dimensions related to self-expressive motives, such as style, bimodal distributions can be partially preferred by consumers. Similar findings are reported by [17].

Research has also explored the interdependencies between various metrics, shedding light on the complex relationships that govern consumer decision-making. For instance, [8] investigate the impact of review volume on consumer preferences under different average rating scenarios. A key finding of their study is that consumers exhibit a preference shift towards products with lower average ratings but a higher number of reviews, as opposed to products with higher average ratings but fewer reviews. Similarly, [18] demonstrate that customers are more sensitive to the mean and volume of ratings, rather than the variance or origin of the ratings, when making purchasing decisions. Furthermore, [19] highlight the importance of average ratings in assessing the quality of the underlying product, although they find that the volume of ratings has a negligible impact on this evaluation.

### Derivation of aggregation functions

The literature on aggregation functions can be broadly categorized into two distinct streams. The first stream of research concentrates on the development and evaluation of novel aggregation functions, with a focus on elucidating their mathematical underpinnings and assessing their potential applicability in diverse reputation systems, often through theoretical analyses or simulation-based approaches (for a comprehensive overview, see [9]). While these studies typically provide in-depth examinations of the theoretical properties of various aggregation functions, they often neglect to investigate how these functions are actually utilized by consumers in real-world settings, thereby leaving a gap in our understanding of their practical implications.

The second stream of research adopts a more empirical approach, focusing on the examination of the arithmetic mean’s usage and its boundary conditions in real-world decision-making contexts. In these studies, participants are typically presented with information on the arithmetic mean and the underlying rating distribution (as illustrated in Fig 1), and experimental conditions are carefully designed to elicit insights into the effectiveness of the arithmetic mean as a decision-support tool. The primary objective of this approach is to investigate whether consumers’ choices and decisions align with the predictions based on the arithmetic mean.

**Fig 2:**
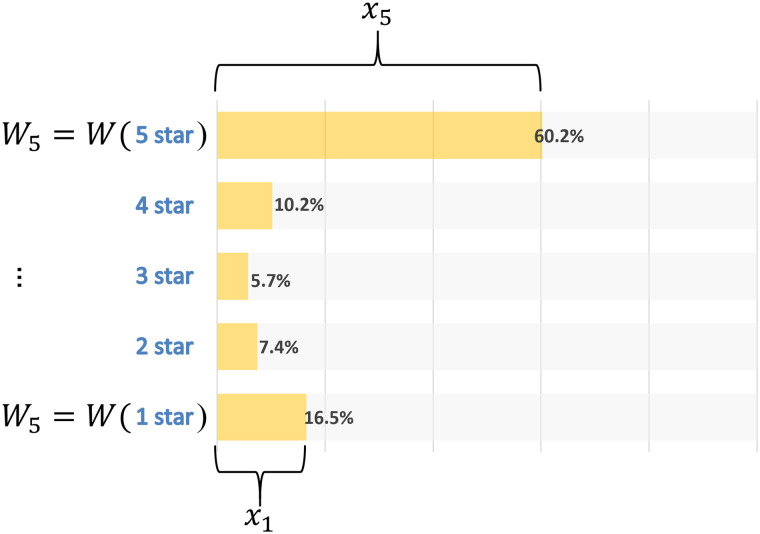
Framework for measuring valence of customer rating distributions with category weights $W_{k}$ and relative frequencies $x_{k}$ (illustrated with an example rating distribution).

Our research agenda occupies a novel position at the intersection of the two existing strands, introducing an innovative distinction: whereas prior studies have primarily focused on assessing the usage of the arithmetic mean, we undertake a more comprehensive approach. Specifically, we derive a plausible set of diverse aggregation functions and investigate the extent to which consumers’ product rankings align with the principles of these alternative aggregation functions. This approach enables us to examine which aggregation functions consumers actually employ when evaluating products based on rating distributions. The derivation of these aggregation functions is grounded in typical numerical rating distributions, which are known to influence consumers’ perceptions of product quality. Our framework captures the valence of a product through a flexible functional form, allowing us to derive different aggregation functions, including the arithmetic mean, by varying the subjective weights that consumers assign to different categories of the rating distribution. More specifically, as illustrated in Fig 2, we consider a typical customer rating distribution comprising categories *k* = 1,...,5 and the relative frequency $x_{k}$ of category *k*. Another interpretation for $x_{k}$ is that it captures the probability that the product’s quality falls into category *k*. The variable $W_{k}$ represents the subjective weight that a consumer attaches to the category *k* with higher weights indicating greater importance in the consumer’s evaluation process of the product’s quality. We model the valence *v* of a product as a function of these category weights and the relative frequencies over all available categories *k*:


$$\begin{array}{c} v=\sum_{k=1}^{5}W_{k}\times x_{k} \end{array}$$ (1)

By systematically varying the category weights, we derive a set of plausible aggregation functions that customers may employ to capture the valence of a product. Whenever possible, we also reference empirical studies that have investigated the importance and relevance of these aggregation functions.

#### Arithmetic mean (AM).

We commence our analysis with the arithmetic mean where the valence score is calculated by weighting the rating categories in proportion to their scale values. The intuitive interpretation, ease of computation, and documented correlation with sales performance (as noted in, for example, [2]) contribute to its widespread adoption. The arithmetic mean uses all available data points in the distribution, assigning each rating a proportional weight. Within our framework, this translates to weighting each category in accordance with its ordinal category scale, such that $W_{k}=k$. Consequently, calculating the valence based on the principles of the arithmetic mean can be expressed as:


$$\begin{array}{c} v_{AM}=\sum_{k=1}^{5}k\times x_{k}. \end{array}$$

#### Focus on the highest category (FIV).

As an alternative to the arithmetic mean, our first approach posits that customers calculate a product’s valence by prioritizing five-star reviews. This assumption is grounded in the prevalence of J-shaped rating distributions in online marketplaces, as noted by [20]. In this context, the resulting aggregation function is approximately equivalent to the mode. By concentrating on 5-star ratings, consumers consider only the most positive experiences, thereby employing a simple decision rule that mitigates cognitive load while potentially capturing the most diagnostic quality information. Within our framework, customers utilize this aggregation function by evaluating only the relative frequency of 5-star ratings in the distribution, while disregarding all other categories, i.e.,: $W_{1}^{FIV},\;...,\;W_{4}^{FIV}=0,\;W_{5}^{FIV}=1$.


$$\begin{array}{c} v_{FIV}=\sum_{k=1}^{5}W_{k}^{FIV}\times x_{k}=x_{5} \end{array}$$

#### Focus on the lowest category (ONE).

Likewise customers may put emphasis on the lowest category to capture the product’s valence. Empirical evidence from [21] demonstrates a shift from unidimensional to multidimensional reputation systems, revealing that the aggregation of sub-dimensions into an overall rating does not proceed via equal-weighted averaging. Instead, the observed higher average ratings in multidimensional systems suggest a cognitive bias toward salient negative attributes, indicating that reviewers tend to overweight adverse experiences when forming overall judgments. This phenomenon is further corroborated by [22], who document a disproportionate impact of extreme negative ratings (i.e., one-star reviews) on consumer perceptions, compared to moderately negative evaluations. Such findings imply that the frequency of one-star ratings serves as a salient heuristic for assessing product quality, particularly in contexts where risk aversion shapes decision-making. In our framework, customers using this aggregation function to calculate the product’s valence assess a customer rating distribution only by the relative frequency of the negative 1-star ratings, i.e.,: $W_{1}^{ONE}=-1;\;W_{2}^{ONE},\;...,\;W_{5}^{ONE}=0$.


$$\begin{array}{c} v_{ONE}=\sum_{k=1}^{5}W_{k}^{ONE}\times x_{k}=-x_{1} \end{array}$$

#### Binary perception of ratings (BIN).

[23] provide evidence for a cognitive simplification pattern they term the binary bias in consumer evaluation of rating distributions. Their findings indicate that individuals tend to categorize ratings in a dichotomous manner: 4-star and 5-star reviews are perceived uniformly as positive, while 1-star and 2-star ratings are aggregated into a single negative category. Ratings on the middle of the scale—particularly 3-star reviews—are frequently interpreted as ambiguous or uninformative, leading to a systematic reduction of the original five-point scale into a three-category framework: positive, neutral (or non-committal), and negative. In our framework, this corresponds to the aggregation function with the following weights: $W_{1}^{BIN}=W_{2}^{BIN}=-1,\;W_{3}^{BIN}=0,\;W_{4}^{BIN}=W_{5}^{BIN}=1$.


$$\begin{array}{c} v_{BIN}=\sum_{k=1}^{5}W_{k}^{BIN}\times x_{k}=-x_{1}-x_{2}+x_{4}+x_{5} \end{array}$$

The next two aggregation functions combine the selective focus on a range of categories and a binary perception of the different categories.

#### Focus on positive ratings (POS).

Empirical evidence suggests that 5-star ratings may not always serve as a reliable indicator of true product quality, as they can be subject to various cognitive biases and strategic manipulation (e.g., [24,25]). Consequently, consumers may adopt a more robust evaluative strategy by incorporating both 5-star and 4-star ratings into their assessment of positive performance. By considering both excellent (5-star) and good (4-star) experiences, consumers consider a broader range of favorable experiences rather than just the most extreme positive reviews. However, as demonstrated by [23], individuals often fail to distinguish between 4-star and 5-star ratings, treating them as functionally equivalent in their evaluative significance. In our framework, this corresponds to an aggregation function that assigns equal weight to both 4-star and 5-star ratings, effectively summarizing the rating distribution through the combined relative frequency of these two categories while disregarding all lower-rated responses, i.e.,: $W_{1}^{POS},\;...,\;W_{3}^{POS}=0;\;W_{4}^{POS}=W_{5}^{POS}=1$.


$$\begin{array}{c} v_{POS}=\sum_{k=1}^{5}W_{k}^{POS}\times x_{k}=x_{4}+x_{5} \end{array}$$

#### Focus on negative ratings (NEG).

Similarly, there is growing evidence that consumers who place heightened emphasis on negative reviews may not rely exclusively on 1-star ratings, particularly due to skepticism regarding the authenticity of online feedback (e.g., [26,27]). Consumers may instead aggregate 1-star and 2-star reviews as a collective indicator of negative sentiment. This strategy reflects a precautionary approach to risk assessment, where the inclusion of both extreme and moderately negative evaluations captures a more comprehensive picture of potential downside risk. Yet, as demonstrated by [23], individuals exhibit limited discriminative capacity between 1-star and 2-star ratings, often treating them as functionally equivalent in their evaluative weight. In our framework, this behavior corresponds to an aggregation function that assigns equal weight to both 1-star and 2-star ratings, summarizing the customer rating distribution through the cumulative relative frequency of these two categories while disregarding all higher-rated responses, i.e.,: $W_{1}^{NEG}=W_{2}^{NEG}=-1;\;W_{3}^{NEG},\;...,\;W_{5}^{NEG}=0$.


$$\begin{array}{c} v_{NEG}=\sum_{k=1}^{5}W_{k}^{NEG}\times x_{k}=-(x_{1}+x_{2}) \end{array}$$

#### Median (MED).

As a final aggregation function, we consider the median, which is defined as the central value in an ordered ranking of all observed ratings. Specifically, the ratings are sorted in ascending order, and the median corresponds to the category that occupies the middle position in this sequence. This approach, as discussed by [28], is robust to extreme values and outliers, as it does not rely on the aggregation of all data points with equal weight, thereby mitigating distortions caused by skewed or anomalous distributions. The median is widely regarded as a particularly reliable and truthful summary statistic, owing to its theoretical properties of minimizing absolute deviations and maximizing robustness under various distributional assumptions [4]. Furthermore, [29] demonstrate that the median outperforms alternative aggregation rules — including the mode, skewness, and kurtosis — in terms of stability and informativeness across diverse rating distributions. [30] similarly advocate for the median as a preferred aggregation mechanism, especially in contexts where rating distributions exhibit significant skewness. Although our modeling framework does not explicitly construct the median in the same operational manner as in traditional statistical aggregation (e.g., by directly computing the middle-ranked category), we nonetheless examine the extent to which consumers’ product selection behavior aligns with median-based evaluation.

We formulate two propositions regarding the adoption of distinct aggregation functions in consumer evaluation. The arithmetic mean remains the industry standard and is widely accepted as a valid and reliable metric for aggregating customer ratings and computing a product’s valence in online marketplaces [29,31]. Empirical studies further indicate that the arithmetic mean is not only statistically well-established but also cognitively familiar to the majority of consumers, making it a salient and frequently used aggregation method in quality assessment [7]. We therefore propose:

**Proposition 1.** *Provided with product rating distributions, the majority of customers apply the arithmetic mean to assess the quality of products.*

However, as detailed in our earlier discussion of alternative aggregation functions, substantial empirical and behavioral evidence indicates that consumers do not consistently adhere to the arithmetic mean as their primary aggregation rule. Instead, they may rely on alternative evaluative strategies, such as selective attention to extreme ratings, heightened sensitivity to negative feedback, or categorical simplification of the rating scale into binary (positive/negative) categories. While our framework does not specify a unique alternative aggregation function nor assume uniform usage across consumers, we acknowledge that such strategies are prevalent. Therefore, we propose that there exists meaningful heterogeneity in the way consumers aggregate rating information:

**Proposition 2.** *Provided with product rating distributions, customers apply heterogeneous aggregation functions to evaluate the quality of products.*

A growing body of literature demonstrates that consumer behavior is sensitive to the format and granularity of information presented in online rating systems, e.g., [32]. For instance, when consumers expect quantitative, detailed data but are instead exposed only to visual summaries, they might change evaluations and hence behave differently [33]. Similarly, [34] find that the perceived valence of product ratings is shaped by the visual representation of rating distributions prior to consumption. When rating information is presented numerically rather than visually, post-consumption ratings tend to be more negative, suggesting that numerical presentations have a stronger impact on consumer judgment. In contrast, [7] report that when only a single bar displaying the mean rating is presented, purchase intention is higher than when full rating distributions are shown, despite identical mean values. This suggests that aggregate metrics, particularly the mean, may be more influential in purchase decisions than distributional information. In conclusion, there is empirical evidence that the amount of information on evaluating product quality can change customers’ purchase decisions. We therefore examine to what extent the adoption of different aggregation functions is affected by the availability of numerical information alongside rating distributions and propose:

**Proposition 3.** *Customers’ applied aggregation principle to judge the quality of products is affected by the availability of provided numerical information.*

## Experimental design

In contrast to the majority of preceding research, which has traditionally assessed consumer behavior in relation to a benchmark behavior predicted by the arithmetic mean, our methodology deviates from this conventional approach. Instead, we employ a novel technique wherein participants are requested to rank diverse customer rating distributions, thereby enabling us to infer their underlying aggregation function(s). This approach is similar to [35] who use comparative data between products to derive ranking of products. This innovative method allows us to examine which aggregation function the average consumer uses and to identify alternatives to the arithmetic mean that consumers may employ in practice.

**Fig 3:**
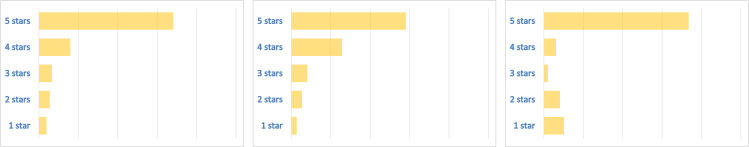
Illustrative bundle of three aggregated customer ratings in the control treatment. Only graphical representations are provided.

The experimental design is implemented as follows: participants are presented with customer ratings for three distinct products and are asked to rank these products in accordance with their preferences. The customer ratings provided differ in terms of their relative frequencies and their arithmetic means. An example of these aggregated customer ratings is depicted in Fig 3.

In this experimental setup, participants are solely presented with the aggregated customer ratings for each product, without being provided with the product’s name or detailed specifications, thereby eliminating potential confounding effects of other product attributes. However, they are informed that the products within each category are comparable in terms of price and specifications. To ensure a controlled environment for evaluation, we carefully select the aggregated customer ratings to facilitate the distinction between disparate aggregation principles, including the minimization of negative ratings, the maximization of positive ratings, and the adherence to the arithmetic mean.

Participants make ranking decisions for a total of 12 product categories. The rating distributions in these categories come from two different sources. Six categories feature aggregated customer ratings from the Amazon marketplace, representing authentic consumer feedback. The remaining six categories contain partially artificial distributions, designed to differentiate between closely related aggregation functions, such as the arithmetic mean versus the median. The complete set of aggregated customer ratings used in the experiment is provided in the S1 Table in the S1 File. At the beginning of the experiment, participants are informed that not all categories are based on real distributions; however, they do not know which specific categories contain artificial ratings. To ensure truthful preference revelation, we employ a carefully designed incentive mechanism. Three product categories are used to determine participants’ payoffs. Each participant receives a USB flash drive based on their ranking decision in one category, either their first or second choice. Additionally, participants have a chance to win an extra product from one of two other categories, either a tablet computer or a gooseneck tablet holder. For the incentivized categories, participants have a 70% chance of receiving their first-ranked product and a 30% chance of receiving their second-ranked product. This incentive structure encourages participants to reveal their true preferences across the entire ranking. The product categories are presented in random order, and participants learn which categories are incentivized only at the end of the experiment.

**Fig 4:**
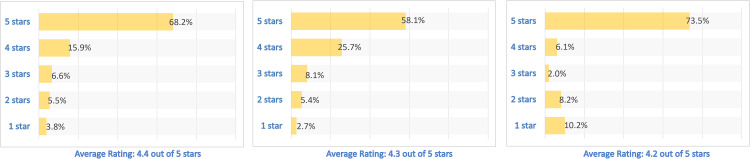
Illustrative bundle of three aggregated customer ratings in the information treatment. Information on the relative frequencies and the arithmetic mean are provided.

We employ two treatments in our experiment. In the *control treatment* (CT), participants are presented with only the graphical representation of aggregated customer ratings without any additional numerical information (as shown in Fig 3). In contrast, the *information treatment* (IT) provides participants with both the graphical representation and the numerical values of the rating distribution, including the relative frequency of each star category and the arithmetic mean (as shown in Fig 4). This treatment variation enables us to test Proposition 3, which suggests that the aggregation principles applied by participants will be influenced by the availability of numerical information.

Following the experiment, participants complete a questionnaire that collects socio-demographic characteristics, risk preferences, experience with online shopping, and self-reported decision criteria used for ranking the products. This information allows us to control for individual characteristics that may impact aggregation behavior.

Participants were recruited via the online recruiting system ORSEE [36], from a pool of approximately 2,800 students from different fields of study who volunteered to become prospective participants in economic decision experiments. The experiment was conducted at the Business and Economic Research Laboratory (BaER-Lab) at Paderborn University, Germany, in December 2018. We conducted four sessions with a total of 107 participants. The experiment was computerized and implemented using the software z-Tree [37]. At the beginning of each session, participants received the same introductory talk and were told not to communicate with each other for the duration of the experiment. Participants then read written instructions and had the opportunity to ask individual questions to the experimenter in private before the experiment began. Once all participants were ready, the experiment was started. Sessions lasted 75 minutes on average. Participants earned material prizes with an average value of EUR 19.10, plus a show-up fee of EUR 2.50.

## Data and statistical model

The data gathered from our laboratory experiment can be summarized in the form of a matrix, separately for the control (CT) and the information treatment (IT) as follows:


$$\begin{array}{c} Z=(\begin{array}{cccc} z_{1,1} & z_{1,2} & \cdots & z_{1,m}\\ z_{2,1} & z_{2,2} & \cdots & z_{2,m}\\ \vdots & \vdots & \ddots & \vdots \\ z_{n,1} & z_{n,2} & \cdots & z_{n,m} \end{array}) \end{array}$$

In this matrix, the rows correspond to the participants (53 in CT and 54 in IT), while the columns represent the product groups (12 in total, identical for both CT and IT). Each element $z_{i,j}$ in the above matrix captures the preference of participant *i* over product group *j*, specifically, the ranking


$$\begin{array}{c} z_{i,j}:\;o_{\pi_{i,j}(1)}≻o_{\pi_{i,j}(2)}≻o_{\pi_{i,j}(3)} \end{array}$$ (2)

of three different products in total order. Here, $\pi_{i,j}$ denotes a permutation {1,2,3}⟶{1,2,3} such that $\pi_{i,j}(k)$ is the index of the object on position *k* in the ranking. Each product $o_{i}$ is characterized by its customer rating distribution, represented as


$$\begin{array}{c} f(o_{i})=(x_{i,1},x_{i,2},x_{i,3},x_{i,4},x_{i,5})\in [0,1{]}^{5}\;, \end{array}$$ (3)

where $x_{i,k}$ is the relative frequency of *k*-star ratings for the product. For the detailed customer rating distributions we refer back to S1 Table in the S1 File.

### Plackett-Luce model

To empirically analyze and test our propositions, a suitable stochastic model of the data-generating process is required. According to our assumptions, this process consists of two stages. When confronted with a set of three choice alternatives, a participant first evaluates each alternative in terms of a (latent) utility degree, and then sorts them in decreasing order of preference according to these degrees. To account for inaccuracies, mistakes, and other random effects, the stochastic nature of the model is clearly important. Since the observational data consists of rankings (2), we employ the so-called Plackett-Luce (PL) model [38,39], which is a model of rank data that is parametrized by quantitative preference degrees for individual choice alternatives. This model nicely complies with the assumptions of our data-generating process.

More specifically, the PL model defines a probability distribution of the set of all rankings of a given set $\left\{o_{1},\ldots ,o_{K}\right\}$ of *K* choice alternatives, that is, on the set of all permutations of [K]={1,…,K}. It is parametrized by a vector $v=(v_{1},v_{2},\ldots ,v_{K})\in {\mathbb{R}}_{+}^{K}$, where each $v_{i}$ can be interpreted as the weight or “strength” of the product option $o_{i}$. The probability assigned by the PL model to a ranking represented by a permutation $\pi \in {𝕊}_{K}$ is given by


$$\begin{array}{c} p_{v}(\pi )=\prod_{i=1}^{K}\frac{v_{\pi (i)}}{v_{\pi (i)}+v_{\pi (i+1)}+\ldots +v_{\pi (K)}}. \end{array}$$ (4)

The mathematical product on the right-hand side of equation (4) represents the probability of generating the ranking π through a *sequential* process. Specifically, the selection process unfolds in a stagewise manner, where the item in the first position is chosen first, followed by the item in the second position, and so on. At each stage, the probability of selecting an item as next choice is proportional to its corresponding weight. Consequently, items with higher weights tend to be assigned to higher-ranking positions. In particular, the most probable ranking (i.e., the mode of the PL distribution) is simply obtained by sorting the items in decreasing order of their weights:


$$\begin{array}{c} \pi^{*}=\underset{\pi \in {𝕊}_{K}}{\mathrm{argmax}}p_{v}(\pi )=\underset{k\in [K]}{\mathrm{argsort}}\{v_{1},\ldots ,v_{K}\}. \end{array}$$ (5)

In our specific context, *K* = 3, $v_{j}$ represents the latent utility of the $j^{th}$ product. For example, π=(π(1),π(2),π(3))=(2,3,1) represents the ranking $o_{2}≻o_{3}≻o_{1}$, indicating that the second product is the most preferred, the third product is the second-best, and the first product is the least preferred. The PL probability of observing this ranking is


$$\begin{array}{c} p_{v}(\pi )=\frac{v_{2}}{v_{1}+v_{2}+v_{3}}\times \frac{v_{3}}{v_{1}+v_{3}}\times \frac{v_{1}}{v_{1}}\;. \end{array}$$

### Product preferences

In the general case, we posit that each product $o_{j}$ can be represented by a set of descriptive statistics (or features) $\left(x_{j,1},\ldots ,x_{j,k}\right)$ that characterize the underlying customer rating distribution. It is reasonable to assume that the utility $v_{j}$ can be modeled as an aggregation of these features:


$$\begin{array}{c} v_{j}=A(x_{j,1},\ldots ,x_{j,n})\;, \end{array}$$

where *A* denotes a suitable aggregation function. Specifically, we assume that the utility $v_{j}$ can be represented as a log-linear function of a *generalized mean*:


$$\begin{array}{c} v_{i}=\exp\left(\alpha \sum_{k=1}^{5}x_{k}\;w_{k}\right)=\exp(\alpha \langle x,w\rangle )\;, \end{array}$$ (6)

where the coefficient $w_{k}$ captures the importance of the *k*-star frequency $x_{j,k}$ (cf. Fig 2).

Since the PL model is invariant to multiplication of the parameter v by a positive constant, and the parameter α>0 accounts for scaling effects, we can normalize the coefficients such that


$$\begin{array}{c} \sum_{k=1}^{5}w_{k}=0\;,\;\sum_{k=1}^{5}|w_{k}|=1\;. \end{array}$$

This formulation enables a straightforward interpretation of the model: The sign of the coefficient $w_{k}$ determines the direction of the influence of the frequency $x_{k}$ on the preference (positive or negative), while the absolute value reflects the relative importance of the *k*-star category compared to the others. The parameter α captures the “precision” of the decision-maker: the larger the value of α, the higher the probability that the generated ranking will align with the latent utilities. In particular, the probability of the mode (5) converges to 1 for α→∞. This case corresponds to a perfect decision maker who deterministically ranks products in accordance with their latent utilities. At the opposite extreme, α=0 leads to a uniform distribution over the set of possible rankings. In other words, α=0 corresponds to a decision maker who effectively ignores the utilities and instead sorts the products completely at random.

The model outlined above imposes a restriction on the class of aggregation functions *A* by assuming that the frequency of ratings is combined through a weighted average (see, e.g., [40] for other classes of aggregation measures). While this assumption may be subject to criticism, it is worth noting that our framework still encompasses a broad range of important aggregation functions as special cases. In particular, the simple arithmetic mean (AM) can be obtained as a special case by the following weights:


$$\begin{array}{c} w_{1}=-\frac{1}{3},\;w_{2}=-\frac{1}{6},\;w_{3}=0,\;w_{4}=+\frac{1}{6},\;w_{5}=+\frac{1}{3}. \end{array}$$ (7)

Similarly, for instance, the “5-star ratio” (FIV) aggregation function can be obtained by


$$\begin{array}{c} w_{1}=-\frac{1}{8},\;w_{2}=-\frac{1}{8},\;w_{3}=-\frac{1}{8},\;w_{4}=-\frac{1}{8},\;w_{5}=+\frac{1}{2}. \end{array}$$

Note that all considered aggregation functions in this study are encompassed by the aggregation function (6) as special cases. Table S2 in the S1 File provides theoretical and estimated weights for each discussed aggregation function. The sole exception is the median aggregation function (MED), which cannot be analytically derived from our framework because the corresponding weights do not depend solely on individual rating categories but rather on the entire customer rating distribution. Specifically, the median identifies the middle value when the relative frequencies of all rating categories are ordered, meaning its calculation requires information about the complete distribution structure rather than just the relative frequencies of individual categories.

### Parameter estimation

The model introduced above is parametrized by two key components: α, which captures the precision of a subject’s decisions, and $w=(w_{1},\ldots ,w_{5})$, which represents the aggregation behavior. Together, these parameters determine the PL parameters (6), which in turn determine the probability of observing the specific rankings (4). Thus, estimating our model reduces to estimating the values of α and w. In this section, we address the estimation problem using the principle of maximum likelihood (ML) estimation.

Assuming that we have observed a dataset 𝒟 consisting of *N* rankings $\pi_{1},\ldots ,\pi_{N}$, where each ranking corresponds to a preferential ordering of three products $o_{1},o_{2},o_{3}$, the likelihood of the parameters α and w can be expressed as the probability of observing this dataset:


$$\begin{array}{c} L(\alpha ,w)=\prod_{n=1}^{N}p_{\alpha ,w}(\pi_{n})=\prod_{n=1}^{N}\prod_{j=1}^{3}\frac{v_{\pi (j)}}{\sum_{i=j}^{3}v_{\pi (i)}}\\ =\prod_{n=1}^{N}\prod_{j=1}^{3}\frac{\exp\left(\alpha \sum_{k=1}^{5}w_{k}\;x_{\pi (j),k}\right)}{\sum_{i=j}^{3}\exp\left(\alpha \sum_{k=1}^{5}w_{k}\;x_{\pi (i),k}\right)}\; \end{array}$$ (8)

where $x_{j}=(x_{j,1},\ldots ,x_{j,5})$ is the frequency distribution of the $j^{th}$ product, and $p_{\alpha ,w}$ the PL probability with parametrization (6). Thus, the ML estimate is obtained as


$$\begin{array}{c} (\alpha^{*},w^{*})={\text{arg max}}_{\alpha ,w}\sum_{n=1}^{N}\sum_{j=1}^{3}(\alpha \sum_{k=1}^{5}w_{k}\;x_{\pi (j),k}\\ -\log(\sum_{i=j}^{3}\exp(\alpha \sum_{k=1}^{5}w_{k}\;x_{\pi (i),k})))\;. \end{array}$$

To show the convexity of the negative log-likelihood function, or equivalently the concavity of (8), we reparametrize the model (6) as follows:


$$\begin{array}{c} v_{i}=\exp\left(\alpha \sum_{k=1}^{5}x_{k}\;w_{k}\right)=\exp\left(\sum_{k=1}^{5}x_{k}\;\alpha w_{k}\right)\\ =\exp(\langle x,\alpha w\rangle )=\exp(\langle x,w^{\prime }\rangle )\;, \end{array}$$ (9)

with


$$\begin{array}{c} \sum_{k=1}^{5}w_{k}^{\prime }=0\;,\;\sum_{k=1}^{5}|w_{k}^{\prime }|=\alpha \;. \end{array}$$

The resulting model is well-established in the preference learning literature and known as the *Plackett-Luce model with features* [41]. As previously shown by [42], the negative log-likelihood of this model is convex. Moreover, the authors proved that the more general (bilinear) Plackett-Luce model is identifiable, which implies that our model is also identifiable. Consequently, the parameter estimation can be accomplished using quasi-Newton type algorithms such as L-BFGS-B [43]. In our setting, we estimate the parameters on the basis of different data sets 𝒟 using three complementary approaches. First, we estimate the weights of the unrestricted Plackett-Luce model, both pooled across the full sample and partially pooled conditional on the two treatments, to derive average aggregation behavior and assess whether it differs across conditions of numerical information. We then compare the estimated weights for similarities and differences with the theoretical weight patterns of the candidate aggregation functions, in particular with the AM. Second, we evaluate model fit by comparing the unrestricted model against restricted versions corresponding to each candidate aggregation function. Third, we adopt a subject-wise approach, where a separate model is fitted for each individual subject. This allows for subject-specific preferences, which are assumed to remain constant across all decisions made by a given subject, but may vary between subjects. The subject-wise estimates serve a dual purpose, as they enable us to assess, through individual-level likelihood ratio tests, how many subjects’ weights are consistent with the AM, and they provide the basis for identifying systematic clusters of alternative aggregation behavior.

## Results

### Descriptive statistics

Demographics of the subjects who participated at the control (CT) or information (IT) treatment are provided in Table 1. There is a slight overrepresentation of female participants in our sample. Nevertheless, consistent with all other individual variables, we observe no significant differences in composition across treatment conditions, suggesting that participant characteristics are evenly distributed between treatment groups.

**Table 1:** Demographic information of participants in control (CT) and information (IT) treatment.

		CT (n = 53)	IT (n = 54)	Total
Male		32%	37%	35%
Age		22.2 (2.7)	22.1 (2.9)	22.1 (2.8)
Studies:	Economics	39.6%	33.3%	36.5%
	Education	39.6%	42.6%	41.1%
	Engineering	7.6%	13.0%	10.3%
	Humanities	11.3%	9.3%	10.3%

For the purposes of our analysis, customer rating distributions are denoted as follows: for each triple of customer rating distributions in a given decision, *AM*_*1*_ refers to the distribution $\left(x_{1},\ldots ,x_{5}\right)$ with the highest arithmetic mean $am_{1}=\sum_{k=1}^{5}k\cdot x_{k,1}$, *AM*_*2*_ the distribution with the second-highest mean *am*_2_, and *AM*_*3*_ represents the distribution with the lowest mean value *am*_3_. Assuming that subjects rank products based on the arithmetic mean, the ranking *[AM*_*1*_*,AM*_*2*_*,AM*_*3*_*]* (i.e., $AM_{1}≻AM_{2}≻AM_{3}$) corresponds to what we term the *reference ranking*.

**Fig 5:**
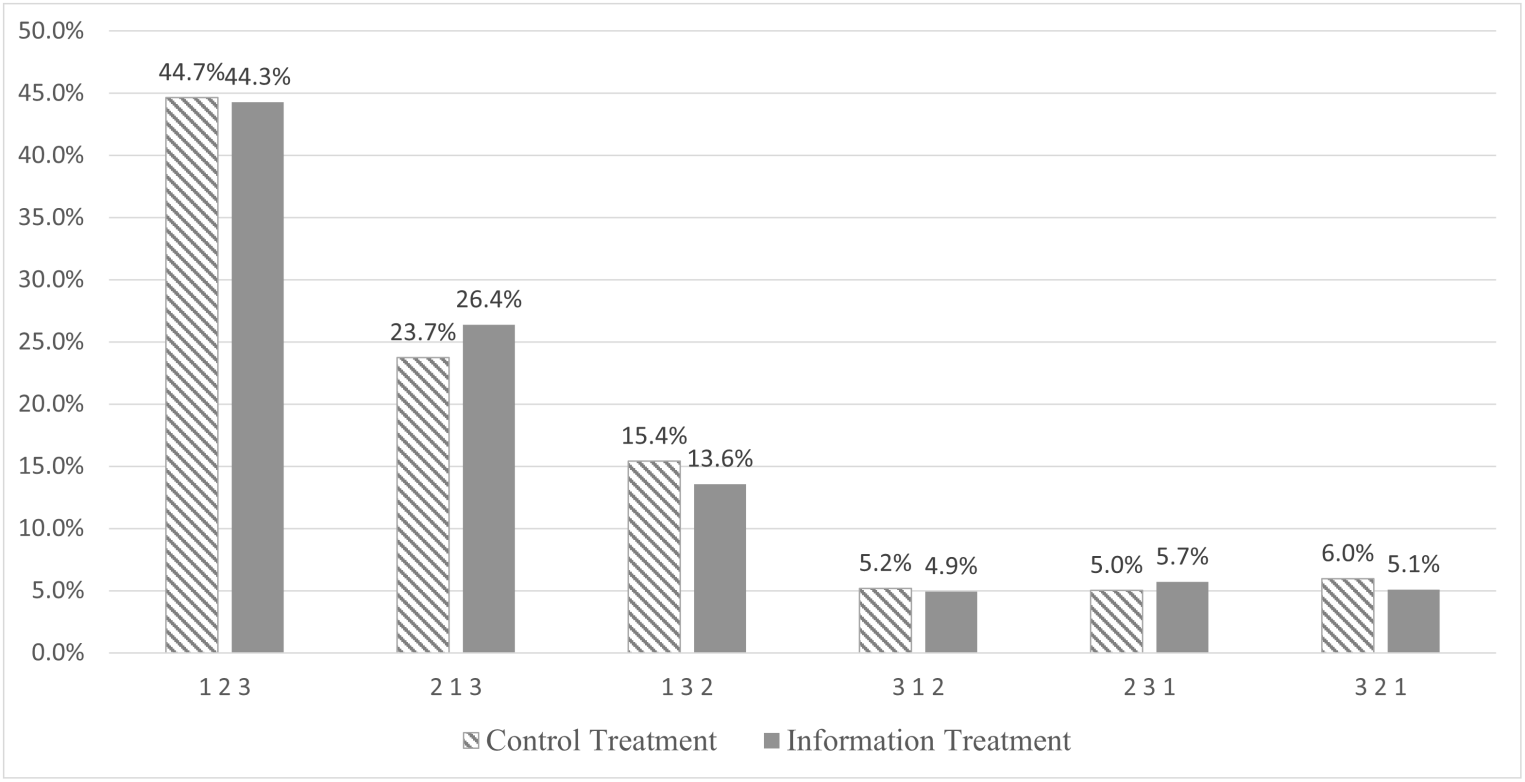
Relative frequency distributions of observed rankings in the control and information treatment. *1* (abbreviation for *AM*_*1*_) is the product with the highest mean, *2* the one with the second-highest mean, and *3* the one with the lowest mean. Hence, *1 2 3* corresponds to the ranking in accordance with the arithmetic mean.

Overall, subjects made a total of 53×12=636 decisions in the control treatment and 54×12=648 decisions in the information treatment. Fig 5 shows the relative frequency distributions over the six possible rankings of all product categories in both treatments.

**Fig 6:**
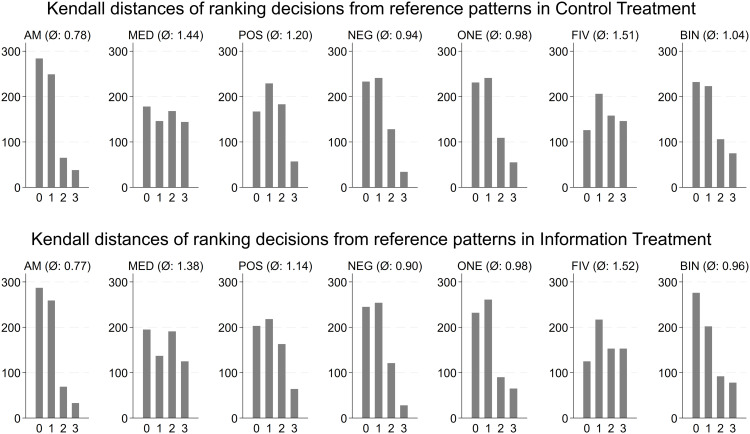
Mean and distribution of Kendall distances (0 = observed ranking fully complies with prediction of aggregation function to 3 = ranking is complete opposite to prediction of aggregation function) for all aggregation functions.

The percentage of decisions in line with the arithmetic mean is nearly identical across treatments, with 44.7% in the control treatment and 44.3% in the information treatment. Detailed ranking decisions over the 12 product categories are listed for both treatments in Table S3 in the S1 File. To measure the distance between the reference ranking *[AM*_*1*_*,AM*_*2*_*, AM*_*3*_*]* and any other ranking, we employ the Kendall distance, which captures the number of pairwise inversions between products, resulting in a distance between 0 and 3. Notably, 83.8% of all decisions in the control treatment and 84.3% in the information treatment are within a distance of at most 1 from the reference ranking (i.e., *[AM*_*1*_*,AM*_*2*_*,AM*_*3*_*]*, *[AM*_*2*_*,AM*_*1*_*, AM*_*3*_*]*, and *[AM*_*1*_*,AM*_*3*_*,AM*_*2*_*]*). Fig 6 provides a more nuanced overview, illustrating the mean Kendall distance and the distribution of Kendall distances (ranging from 0 to 3) for each treatment and aggregation function that we derived from our framework. As indicated earlier, a distance of 0 means that the observed ranking fully aligns with the respective aggregation function, whereas a distance of 3 indicates that the observed ranking is completely reversed compared to what the aggregation function would have predicted. The figure reveals that the arithmetic mean exhibits the lowest mean Kendall distance compared to all other aggregation functions, as well as the highest proportions of 0 and 1 deviations from the predicted ranking. In contrast, the median (MED) and the 5-star focus (FIV) aggregation functions perform worst, as evidenced by their highest average Kendall distances and the dispersed pattern of empirical deviations from the predicted rankings.

In Table 2, we present a comparative analysis of the observed ranking behavior across the different aggregation functions. We hereby provide win/tie/loss statistics. In this comparison, each pair of aggregation functions is compared to every observed ranking using the Kendall distance. The function with the smaller distance is considered the “winner,” while the other is the “loser.” For example, in the top panel that shows the comparisons of the control treatment, the arithmetic mean (AM) is evaluated against the aggregation function of focusing on the highest category (FIV) in the first row and second column. Out of the 636 observed rankings, the AM aggregation function had a lower Kendall distance than FIV in 308 rankings, indicating that the rankings were more consistent with the AM than FIV principles. Conversely, FIV had a lower Kendall distance in 42 rankings, while the two functions were tied in the remaining 286 observed rankings. Notably, the AM aggregation function exhibits a greater number of wins than losses in all pairwise comparisons across both treatments. This finding, combined with the statistics presented in Fig 6, provides initial evidence in favor of the AM aggregation function.

**Table 2:** Win/tie/loss statistics for the different aggregation functions, separately for the control (CT, top panel) and information treatment (IT, bottom panel).

	AM	FIV	ONE	BIN	POS	NEG	MED
AM	0 / 636 / 0	308 / 286 / 42	191 / 330 / 115	243 / 231 / 162	200 / 372 / 64	184 / 317 / 135	237 / 371 / 38
FIV	42 / 286 / 308	0 / 636 / 0	207 / 28 / 401	157 / 134 / 345	144 / 194 / 298	181 / 64 / 391	150 / 270 / 216
ONE	115 / 330 / 191	401 / 28 / 207	0 / 636 / 0	229 / 204 / 203	263 / 198 / 175	121 / 403 / 112	326 / 127 / 183
BIN	162 / 231 / 243	345 / 134 / 157	203 / 204 / 229	0 / 636 / 0	265 / 212 / 159	155 / 265 / 216	227 / 309 / 100
POS	64 / 372 / 200	298 / 194 / 144	175 / 198 / 263	159 / 212 / 265	0 / 636 / 0	158 / 227 / 251	265 / 271 / 100
NEG	135 / 317 / 184	391 / 64 / 181	112 / 403 / 121	216 / 265 / 155	251 / 227 / 158	0 / 636 / 0	330 / 143 / 163
MED	28 / 371 / 237	216 / 270 / 150	183 / 127 / 326	100 / 309 / 227	100 / 271 / 265	163 / 143 / 330	0 / 636 / 0
AM	0 / 648 / 0	314 / 298 / 36	187 / 343 / 118	229 / 239 / 180	182 / 396 / 70	179 / 333 / 136	242 / 378 / 28
FIV	36 / 298 / 314	0 / 648 / 0	198 / 33 / 417	147 / 144 / 357	134 / 193 / 321	179 / 73 / 396	132 / 284 / 232
ONE	118 / 343 / 187	417 / 33 / 198	0 / 648 / 0	221 / 196 / 231	248 / 219 / 181	118 / 396 / 134	329 / 134 / 185
BIN	180 / 239 / 229	357 / 144 / 147	231 / 196 / 221	0 / 648 / 0	274 / 216 / 158	169 / 270 / 209	232 / 320 / 96
POS	70 / 396 / 182	321 / 193 / 134	181 / 219 / 248	158 / 216 / 274	0 / 648 / 0	169 / 235 / 244	270 / 278 / 100
NEG	136 / 333 / 179	396 / 73/ 179	134 / 396 / 118	209 / 270 / 169	244 / 235 / 169	0 / 648 / 0	332 / 150 / 166
MED	28 / 378 / 242	232 / 284 / 132	185 / 134 / 329	96 / 320 / 232	100 / 278 / 270	166 / 150 / 332	0 / 648 / 0

### Aggregation behavior derived from statistical model

We employ several complementary analyses to derive the ranking behavior from our statistical model, progressively moving from pooled estimation to individual-level inference. First, we estimate the weights of the pooled unrestricted Placket-Luce model. The results are reported in Table 3. The estimated weights closely mirror the pattern predicted by the theoretical AM aggregation function (see Table 6). The estimated weights increase monotonically from *w*1 to *w*5 and *w*3 is the only weight that is not significantly different from zero, which is consistent with the theoretical AM value of zero for the middle category. Notably, of the unrestricted model *w*4 is over- and *w*5 is under-weighted compared to the AM weight counterparts, suggesting that on average subjects assign relatively more importance to 4-star ratings and less to 5-star ratings than the AM would predict. In addition, we estimate a partial-pooling version conditional on the two treatments CT and IT. The results, reported in Table 4, are very similar to the pooled ones and across treatments, indicating that the structure of aggregation behavior is robust to the availability of numerical information. Second, to assess which candidate aggregation function best approximates the pooled weights, we re-estimate the model under the restriction that the weights follow the rules of each candidate aggregation function (with the exception of the median aggregation function). The results are shown in Table 5. Based on the conventional metrics of Akaike’s Information Criterion (AIC) and Bayesian Information Criterion (BIC), the AM aggregation function shows the smallest loss of fit relative to the unrestricted model, indicating that the AM aggregation function best describes the data compared to all other candidate aggregation functions. We note, however, that a formal likelihood ratio test rejects the null hypothesis that the pooled weights are equal to the theoretical AM weights ($\chi^{2}(3)=167.69$, *p* < .001). The results indicate that the fixed weighting scheme assumed by the AM model does not fully describe participants’ decisions, as allowing the category weights to vary freely significantly improved model fit. Nevertheless, the strong structural similarity between the pooled weights and the theoretical AM pattern suggests that the AM largely captures the dominant tendency in the data. The rejection of equality likely reflects heterogeneity in individual aggregation behavior that, when aggregated across the full sample, prevents all the pooled weights from converging fully to the AM values.

**Table 3:** Category weight estimates from the pooled unrestricted model (all subjects).

Variable	Coefficient	Std. Err.	z	*p*-value	95% CI	
					Lower	Upper
*w*_1_	−0.3232	0.0065	−49.87	0.000	−0.3359	−0.3105
*w*_2_	−0.1729	0.0082	−21.02	0.000	−0.1891	−0.1568
*w*_3_	−0.0039	0.0060	−0.64	0.521	−0.0157	0.0080
*w*_4_	0.2121	0.0032	67.04	0.000	0.2059	0.2183
*w*_5_	0.2879	0.0032	91.01	0.000	0.2817	0.2941

**Table 4:** Category weight estimates from the partial-pooled unrestricted model by treatment (CT and IT).

Variable	CT Treatment		IT Treatment	
	Coefficient	Std. Err.	Coefficient	Std. Err.
*w*_1_	−0.3290	0.0098	−0.3147	0.0091
*w*_2_	−0.1565	0.0126	−0.1853	0.0091
*w*_3_	−0.0145	0.0088	0.0056	0.0081
*w*_4_	0.2102	0.0047	0.2115	0.0058
*w*_5_	0.2898	0.0047	0.2830	0.0059

All coefficients except *w*_3_ are statistically significant at *p* < 0.001.

**Table 5:** Comparison of models based on Akaike’s Information Criterion (AIC) and Bayesian Information Criterion (BIC).

Model	N	ll(model)	k	AIC	BIC
unrestricted	1,368	−1932.86	4	3873.71	3894.60
AM	1,368	−2016.70	1	4035.40	4040.62
FIV	1,368	−2450.91	1	4903.82	4909.04
ONE	1,368	−2193.06	1	4388.12	4393.34
BIN	1,368	−2122.84	1	4247.67	4252.89
POS	1,368	−2376.20	1	4754.40	4759.62
NEG	1,368	−2198.88	1	4399.75	4404.97

To account for such heterogeneity, we fit in a third step our statistical model in a subject-wise manner. Each model is estimated using |𝒟|=12 ranking instances, with each instance consisting of three products. The preferences of the $i^{th}$ subject are characterized by the corresponding parameter estimate $w_{i}^{*}$. The estimated preference vectors, together with the precision parameter $\alpha_{i}$, are reported in Tables S8 and S9 in the S1 File.

**Fig 7:**
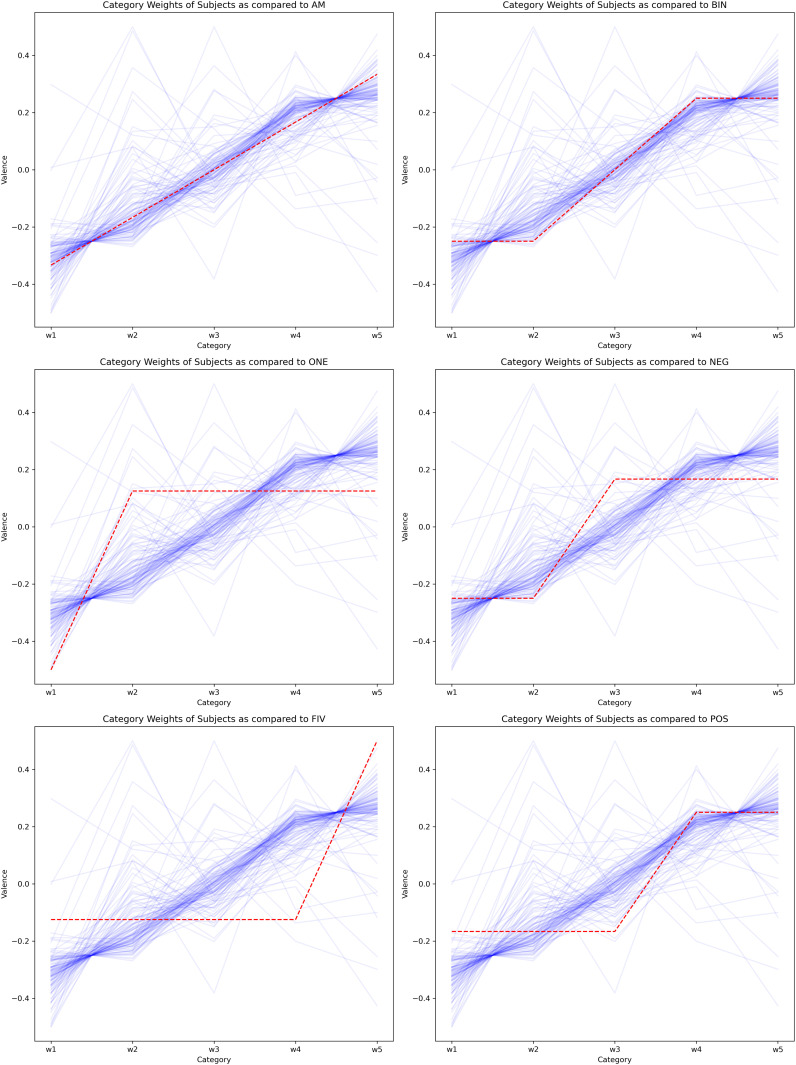
Estimated category weights at the individual subject level. Each solid line represents one subject’s category weights estimated via our maximum likelihood approach. Dotted lines depict the theoretical patterns predicted by the reference aggregation functions.

Fig 7 provides a graphical representation of these preference vectors, alongside the theoretical patterns predicted by the reference aggregation functions discussed above.

Table 6 shows the averaged preferences across both treatments alongside the theoretical and estimated model parameters of the AM aggregation function. The weights and the precision parameter α are very similar across treatments. Moreover, the estimated AM weights fall within the range of the subject-level estimates in both treatments, indicating that the AM pattern is well-represented among the individual-level weight distributions. To assess whether extreme values drive our findings, we exclude subjects with extremely high α parameters (top 10% of the distribution), reducing the sample in each treatment to *n* = 48. As shown in Table 6, the estimated weights change only marginally, indicating that our findings are robust and not driven by outliers in the precision parameter.

**Table 6:** Summary statistics of the estimated model parameters in both CT and IT. Theoretical and estimated parameters of the AM aggregation function.

	*w*_1_	*w*_2_	*w*_3_	*w*_4_	*w*_5_	α
CT	−0.312±.096	−0.113±.139	−0.015±.105	0.189±.075	0.252±.127	266.3±823.6
IT	−0.302±.104	−0.119±.155	0.018±.105	0.177±.100	0.226±.147	236.6±623.8
CT (without extremes)	−0.306±.096	−0.115±.140	−0.024±.105	0.191±.075	0.253±.133	73.1±60.1
IT (without extremes)	−0.304±.110	−0.108±.161	0.017±.109	0.173±.105	0.222±.155	91.1±82.4
Theoretical AM	−0.333	−0.167	0.000	0.167	0.333	
Estimated AM	−0.388	−0.112	0.033	0.138	0.329	

For parameter α, the median (IQR; 90th percentile) was 58.7 (42.6–100.8; 328.7) in the CT treatment and 69.6 (39.7–191.8; 370.8) in the IT treatment. Robustness check: Excluding subjects with extreme α values (top 10% of the distribution), setting the filter at α<358.1, resulting in *n* = 48 in IT and *n* = 48 in CT.

We examine more closely the relationship between individual-level behavior and the behavior predicted by the AM aggregation function by conducting likelihood ratio tests comparing each subject’s unrestricted weights against the estimated AM weights. As reported in Table S11 in the S1 File, for between 54% and 69% of subjects (depending on whether a 1% or 5% significance level is applied) the likelihood ratio test does not reject the null hypothesis that their individual weights are equal to the estimated AM weights. This result provides direct evidence that the AM weights do not accidentally show similarities with the weights from the estimated unrestricted model and merely provide a statistical artifact of averaging across heterogeneous subjects, but reflect the actual aggregation strategy of the majority of individuals. As further corroboration, Table 7 presents, separately for each treatment, the average Kendall distance between the observed product rankings and the rankings predicted by each aggregation function, as well as those predicted by our fitted model. As expected, the estimated model achieves the lowest average distance due to its greater flexibility. However, the AM aggregation function outperforms all other reference aggregation functions, as evidenced by its minimal Kendall distance, and is only slightly surpassed by the fitted model. These results are consistent with the findings from all prior analyses.

**Table 7:** Average Kendall distance for the different aggregation functions and the estimated model, separately for control (CT) and information (IT) treatment.

	AM	FIV	ONE	BIN	POS	NEG	MED	estimated model
CT	0.775	1.509	0.981	1.038	1.204	0.942	1.437	0.541
IT	0.765	1.515	0.981	0.957	1.136	0.895	1.380	0.495

Turning to the goodness of fit of our model, we follow two approaches. First, we examine how well the model predicts rankings that were not used in estimating the model’s parameters. Table 8 reports the out-of-sample prediction performance under five alternative cross-validation schemes. As a benchmark, a random ranking model would assign a probability of 1/6 = 0.167 to the observed ranking. In an initial step, we estimated the model on the six product groups with artificial distributions and used it to predict the rankings of the six product groups with real distributions, and vice versa (Artificial vs. Real). The predictive performance of this cross-distribution validation is poor, with a average fold-wise mean probability of 0.10 and an average fold-wise median of 0.07, both below the random-choice benchmark. This outcome is not surprising given that the two sets of distributions differ substantially in their structural properties. The real distributions, sourced from the Amazon marketplace, predominantly exhibit the J-shaped pattern characteristic of online rating systems, with a concentration of ratings in the higher categories. The artificial distributions, by contrast, were specifically designed to better discriminate between closely related aggregation functions and therefore feature distributional properties that rarely occur in natural online environments. These structural differences mean that a model estimated on one type of distribution is not well-calibrated to predict behavior under the other. We therefore adopted a more appropriate validation strategy by applying leave-one-out procedures separately within each class of distributions. When estimating the model on five of the six real product groups and predicting the held-out group (Real only), the average fold-wise mean probability rises to 0.40, with an average fold-wise median of 0.35. A comparable level of performance is observed for the artificial distributions (Artificial only: mean 0.38, median 0.30). Both schemes substantially exceed the random-choice benchmark, indicating moderate predictive power within each distributional class. When applying a leave-one-out procedure across all twelve product groups (11-vs-1), the mean probability of 0.39 and median of 0.33 confirm that the model generalizes consistently across individual tasks. The somewhat lower performance of the 10-vs-2 scheme (mean 0.31, median 0.24) reflects the reduced estimation basis when two product groups are held out simultaneously. Taken together, these results suggest that the model’s predictive performance is stable and not an artifact of overfitting, provided that the estimation and prediction sets share comparable distributional properties. Second, as detailed in the Supporting Information Appendix (see in particular Tables S10 and S11 in S1 File), we assess model fit indirectly by comparing our approach to alternative methodological frameworks. Based also on these comparisons, we have strong confidence that the goodness-of-fit measure is sufficiently high to ensure the validity of our results. Summarized, with the evidence from earlier analyses, these findings provide clear support for Proposition 1:

**Table 8:** Out-of-sample prediction performance under alternative cross-validation schemes.

Validation scheme	Folds	Mean probability	Median probability	Interpretation
Artificial vs. Real	2	0.10	0.07	Poor transferability
Real only	6	0.40	0.35	Moderate predictive power
Artificial only	6	0.38	0.30	Moderate predictive power
11-vs-1	12	0.39	0.33	Stable across tasks
10-vs-2	6	0.31	0.24	Reduced generalization

Entries report average out-of-sample probabilities assigned to the observed ranking. A random-choice benchmark equals 1/6 = 0.167. Higher values indicate better predictive performance.

**Result 1** *When presented with product rating distributions, the aggregation behavior of the majority of customers in assessing product quality is best characterized by the arithmetic mean.*

### Heterogeneity in aggregating product information distributions

While the preceding analyses establish that the AM best characterizes average aggregation behavior, the subject-level likelihood ratio tests also reveal that a substantial share of subjects deviates significantly from the AM weights. We now examine whether these departures can be attributed to customers’ systematic use of specific alternative aggregation functions. We first examine whether departures from the arithmetic mean are related to characteristics of the product choice sets. Indeed, as shown in Table S3 in the S1 File, the share of subjects following the principles of the arithmetic mean varies across product categories, ranging between 14.8% in product group four and 83.3% in product group eleven (see the R1 shares in the table). To explore whether this variation is systematically linked to properties of the rating distributions, Table S4 in the S1 File reports key characteristics of each product group, including the means and standard deviations of the three products, the difference between the highest and lowest mean, the corresponding difference in standard deviations, and the decision valence, alongside the share of AM-consistent rankings in both treatments. Table S5 in the S1 File reports Spearman correlations between these metrics and the share of subjects following the AM principles. While the direction of the correlations is qualitatively consistent across treatments, with dispersion negatively and decision valence positively associated with AM-consistent rankings, none of the correlations reach statistical significance at conventional levels (all *p* > 0.05). The strongest correlation is observed between the dispersion of ratings and AM-consistent rankings in the information treatment (Spearman’s ρ=−0.514, *p* = 0.087), suggesting a tendency for subjects to depart from AM principles when ratings are more dispersed, though this effect remains marginally significant. These patterns suggest that deviations from the arithmetic mean are not random but follow directionally plausible relationships with the characteristics of the underlying rating distributions. However, the absence of statistically significant correlations, neither separately for each treatment nor pooled, reinforces the need to move to a subject-level analysis, where we can draw on each individual’s full set of 12 ranking decisions to identify persistent aggregation strategies.

In particular, we assess each customer’s 12 ranking decisions against all the aggregation functions discussed above. We then assign each customer to the aggregation function that yields the lowest Kendall distance, as this function most accurately captures the customer’s ranking behavior. The outcome of this sorting procedure is presented in Table 9.

**Table 9:** Clusters of customers assigned to reference functions based on minimal overall Kendall distances (with and without ties).

	AM	FIV	ONE	BIN	POS	NEG	MED
n (with ties, total = 116)	56	4	15	19	3	19	0
% Control Treatment (with ties)	54%	50%	27%	47%	67%	53%	–
% Information Treatment (with ties)	46%	50%	73%	53%	33%	47%	–
n (without ties, total = 99)	51	3	12	17	2	14	0
% Control Treatment (without ties)	51%	67%	33%	53%	100%	43%	–
% Information Treatment (without ties)	49%	33%	67%	47%	0%	57%	–

**Fig 8:**
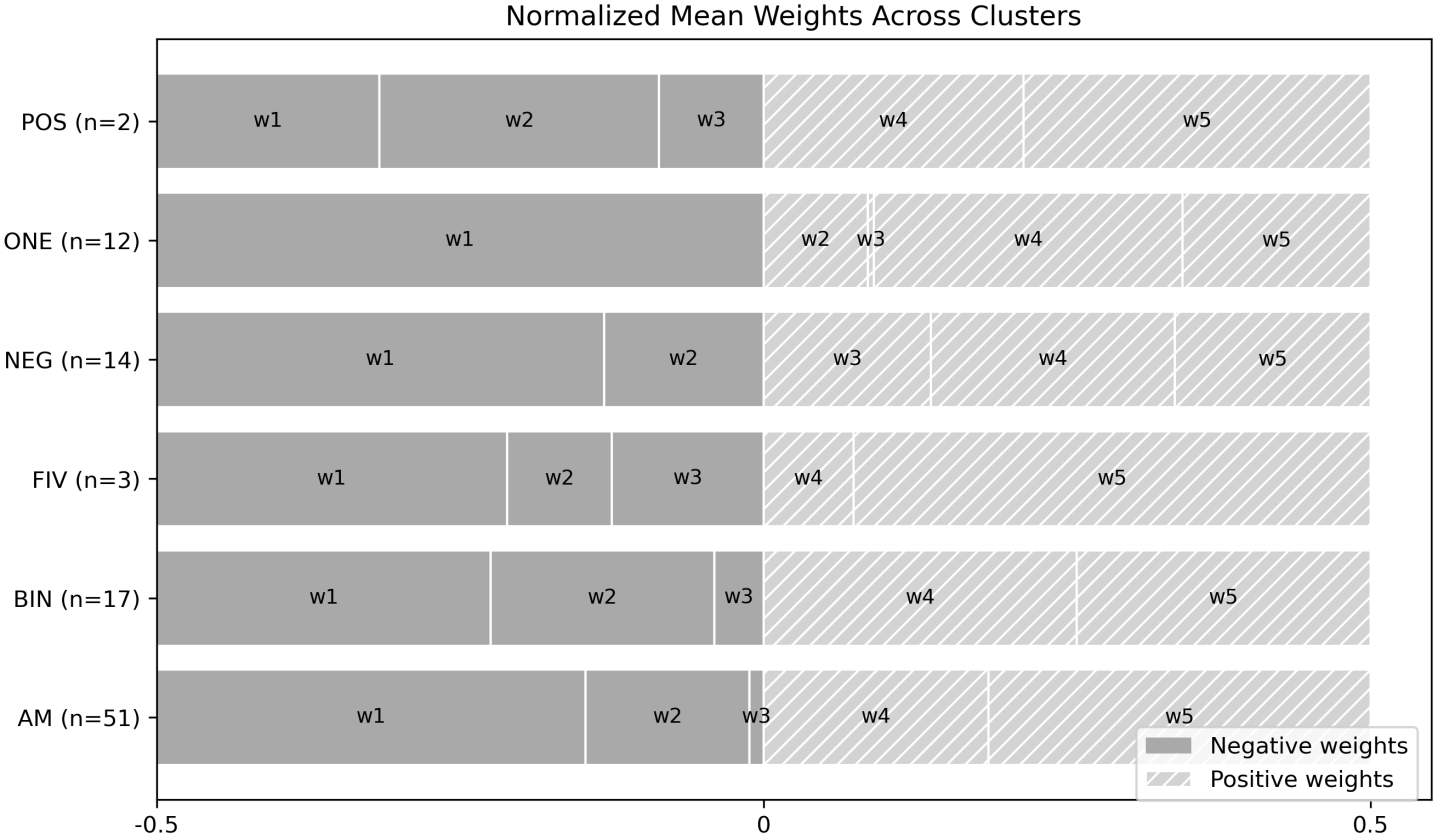
Normalized mean category weights across clusters. Reported are mean weights of subjects within clusters (without subjects whose assignment to clusters are ambiguous). For improved illustration, these mean weights are normalized in a way that absolute category weights add up to 1.

Note that Table 9 reports the outcome of the sorting procedure, both with and without ties. The statistics with ties assigns the same customer to multiple aggregation functions when these functions yield identical overall minimal Kendall distances. In contrast, the statistics without ties include only customers whose minimal overall Kendall distance uniquely identifies a single aggregation function. As shown, the distribution changes only marginally between the two approaches. To validate the accuracy of our sorting procedure, we determine normalized mean weights for each customer cluster that are displayed in Fig 8. The numerical distributions of the estimated category weights for all aggregation functions, including those with and without ties, are reported in the S6 and S7 Tables in the S1 File. Comparison of these estimated weights with the theoretical ones described in the Supporting Information Appendix (S2 Table in S1 File) reveals substantial congruence. For instance, the binary perception of ratings (BIN) exhibits, as expected from theory, similar weights for 1–2 and 4–5 star categories. The estimated weight of 1-star ratings is highest for the customer cluster assigned to the aggregation function that focuses on 1-star ratings (ONE), while the estimated weight of 5-star ratings is highest for the cluster assigned to the FIV aggregation function. Thus, the observed congruence between empirical and theoretical weights provides additional confirmation that customers are accurately classified into their respective aggregation functions. Consistent with our previous analyses, we find that the majority of customers are assigned to the arithmetic mean (AM) aggregation function, indicating that these customers systematically employ arithmetic mean principles to aggregate product information distributions. However, this cluster only accounts for approximately half of the customers. The remaining half appears to aggregate rating distributions based on different principles. First, we identify a substantial cluster of subjects who follow the aggregation principles of the binary perception of ratings (BIN), focusing on both 1–2 star and 4–5 star ratings while discounting the middle category. Second, aggregation behaviors that emphasize negative ratings are prominently represented. The aggregation functions that focus only on 1-star ratings (ONE) and 1–2 star ratings (NEG) together account for another substantial share of customers. Collectively, these alternative clusters to the arithmetic mean account for more than 40% of all participants. Interestingly, we do not find any customers who systematically aggregated product rating information based on the principles of the median (MED).

Despite this finding, we find clear support for Proposition 2, which posits heterogeneity in the use of different aggregation functions. While roughly half of the customers aggregate rating information based on the principles of the arithmetic mean, more than 40% employ aggregation functions with distinct emphases: they either follow a binary approach that equally weights both negative and positive rating categories, or they focus predominantly on negative ratings. We summarize this finding as follows:

**Result 2** *Approximately half of customers aggregate product rating distributions according to the principles of the arithmetic mean. However, a substantial proportion (more than 40%) employs alternative aggregation principles: significant clusters of customers focus exclusively on 1-star ratings, or on 1–2 star ratings collectively, or follow binary patterns in which 1–2 star and 4–5 star rating categories are weighted equally.*

### Impact of numerical information

**Fig 9:**
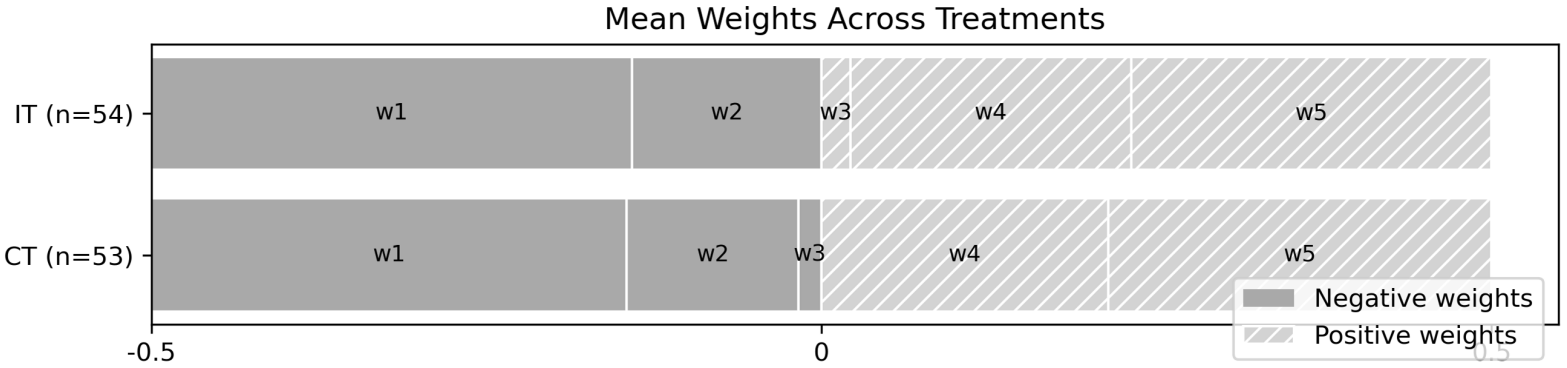
Normalized mean weights $w_{1},\;...,\;w_{5}$ across treatments. Mean weights are derived from participants within treatment groups. For improved visualization, the weights are normalized such that the sum of absolute category weights equals 1 within each treatment.

In both treatments, participants were presented with a graphical representation of the rating distribution for each product. The key distinction between the control and information treatment lay in the provision of numerical information regarding the relative share of each rating category and the explicit statement of the average rating for each product in the latter. An examination of the tabular and graphical summaries from our preceding analyses, which differentiated the results by treatment, reveals that the treatment groups exhibit only negligible discrepancies, suggesting a relatively homogeneous response pattern across treatments. For instance, Table 7 indicates minimal differences in the average Kendall distances for each aggregation function and estimated model across treatments, implying that customers’ aggregation behavior is largely invariant to the amount of numerical information provided. Similarly, Table 9 shows that the distribution of customer clusters assigned to different aggregation functions is only marginally different across treatments, suggesting a consistent systematic use of aggregation functions regardless of treatment. To further assess treatment effects, we estimate the average weight customers placed on each rating category based on the ranking decisions in the respective treatment. The results are displayed in Fig 9. If the provision of numerical information had a substantial impact on aggregation behavior, we would expect to observe significant differences in the distribution of category weights across treatments. However, the results of a multivariate analysis of variance (MANOVA) indicate that the treatment groups do not differ significantly (Wilks’ lambda = 0.9692, p = 0.5214).

Combining the result of this test with statistics already provided in the preceding analyses, we have to reject Proposition 3 and conclude that there is no empirical evidence that aggregation behavior is affected by the availability of additional numerical information.

**Result 3** *Overall, the provision of additional numerical information does not significantly influence customers’ patterns of aggregating product rating distributions.*

To validate the robustness of our findings and assess potential influences of individual characteristics or other contextual factors on customers’ aggregation behavior, we conducted standard multivariate OLS (Ordinary Least Squares) analyses and compared post-experimental questionnaire responses with observed decision patterns.

Table 10 reports the OLS regression results. As the dependent variable, we use for each aggregation function under consideration the aggregated Kendall distance per individual (’sumkdist’) with respect to the respective aggregation function. Negative values of the aggregated Kendall distance indicate a closer alignment between the individual’s ranking decisions and the predictions of the respective aggregation function. Corroborating our preceding analyses we do not see any impact of numerical information on the respective Kendall distances (’Treatment’). Almost all individual or socio-demographic characteristics such as age, gender or field of studies do not exhibit systematic or robust effects on aggregation behavior in any of the different model specifications. The only exception is risk-taking behavior, where we find significant relationships with regard to the aggregation functions that focus on 5-star ratings (FIV), 1-star ratings (ONE), and 1–2 star ratings (NEG). The negative coefficient for FIV indicates that more risk-seeking individuals rank more closely in line with the FIV aggregation function, while the positive coefficients for ONE and NEG suggest that they depart further from the principles of these negative-focused aggregation functions. This pattern is consistent with the interpretation that risk-seeking individuals are less concerned with downside risk and therefore place less emphasis on negative ratings when evaluating products. With regard to shopping experience, the multivariate analysis reveals no statistically significant effect, indicating that in our study shopping experience did not have a substantial impact on ranking decisions. In addition, we include dummy variables derived from the post-experimental questionnaire in which subjects self-reported which aggregation principle they followed when ranking the products. While most self-reported variables show no significant association with actual ranking behavior, the few significant coefficients reveal an interesting pattern between stated and observed decision strategies. For instance, subjects who self-reported following the arithmetic mean show significantly closer alignment not with the AM but with the POS aggregation function. Similarly, subjects who stated they focused on 1-star ratings rank significantly closer to the principles of the AM, and those who reported focusing on positive reviews align more closely with both the BIN and POS aggregation functions. Furthermore, subjects who reported following no particular pattern depart significantly further from the NEG aggregation function. Overall, self-reported aggregation behavior is largely not significantly associated with actual ranking decisions as captured by the Kendall distances, suggesting that subjects’ stated decision strategies do not reliably predict their observed aggregation behavior. In summary, our additional analyses uncover plausible, contextually meaningful relationships. However, with the exception of risk preferences, the results suggest that none of the individual characteristics had a substantial impact on aggregation behavior, reinforcing the validity and robustness of our core findings.

**Table 10:** Multivariate analysis using OLS regression.

	(1)	(2)	(3)	(4)	(5)	(6)	(7)
	sumkdist_AM	sumkdist_FIV	sumkdist_ONE	sumkdist_BIN	sumkdist_POS	sumkdist_NEG	sumkdist_MED
Treatment: IT	0.0368	0.120	0.0214	−0.976	−0.605	−0.302	−0.844
	(0.989)	(0.793)	(0.686)	(0.791)	(0.742)	(0.736)	(0.827)
Age	−0.0174	0.116	0.176	0.0114	−0.0968	0.0772	0.0321
	(0.198)	(0.159)	(0.142)	(0.152)	(0.121)	(0.166)	(0.138)
Studies: Education	−0.792	0.992	−1.546	−0.600	−0.828	−0.274	−0.210
	(1.121)	(0.812)	(0.782)	(0.939)	(0.864)	(0.927)	(0.888)
Studies: Engineering	3.661	1.808	1.194	1.259	1.303	2.253	0.744
	(2.208)	(1.465)	(1.438)	(1.025)	(1.207)	(1.271)	(1.443)
Studies: Humanities	−2.364	−1.823	−0.577	−0.379	−0.668	−0.212	−1.342
	(1.557)	(1.854)	(1.103)	(1.268)	(1.096)	(1.211)	(1.280)
Gender: Male	1.088	0.594	−0.223	−1.275	−0.337	−1.085	0.00696
	(1.180)	(0.884)	(0.765)	(0.904)	(0.820)	(0.914)	(0.870)
Semester	−0.191	−0.185	−0.0776	0.0221	−0.0301	0.0197	−0.182
	(0.177)	(0.131)	(0.146)	(0.125)	(0.119)	(0.122)	(0.130)
General Risk Attitude	−0.154	−0.477*	0.300*	0.316	−0.260	0.375*	−0.317
	(0.210)	(0.215)	(0.145)	(0.170)	(0.136)	(0.148)	(0.171)
Shopping Experience	−1.078	−1.148	0.0150	0.407	0.0674	0.587	−0.435
	(1.158)	(0.981)	(0.720)	(0.779)	(0.750)	(0.771)	(0.918)
Self-reported: ArithmeticMean	−1.804	−1.630	0.127	−1.046	−1.587*	−0.559	−1.482
	(1.184)	(0.890)	(0.737)	(0.798)	(0.774)	(0.804)	(0.906)
Self-reported: Median	−0.157	1.696	−0.652	0.677	0.913	−0.129	1.073
	(1.546)	(1.481)	(0.852)	(0.995)	(1.019)	(0.899)	(1.344)
Self-reported: FiveStars	−1.394	−1.441	0.1000	0.899	−0.619	0.295	−0.455
	(1.205)	(1.055)	(0.910)	(1.160)	(0.983)	(1.096)	(1.074)
Self-reported: OneStar	−2.758*	−1.184	−0.704	−1.331	−0.957	−0.938	−1.152
	(1.116)	(1.095)	(0.870)	(1.078)	(0.936)	(1.094)	(1.149)
Self-reported: NegativeReviews	0.704	1.562	−0.752	0.382	0.564	−0.390	0.893
	(1.137)	(0.849)	(0.922)	(0.943)	(0.811)	(1.045)	(0.766)
Self-reported: PositiveReviews	−0.999	0.381	−0.147	−2.165*	−1.766*	−0.739	−1.361
	(1.108)	(0.837)	(0.868)	(0.974)	(0.811)	(1.034)	(0.836)
Self-reported: NoPattern	−1.091	−4.736	2.939	1.273	−1.613	4.217**	−3.589
	(2.056)	(3.990)	(1.790)	(2.408)	(1.344)	(1.589)	(2.003)
Constant	15.11**	20.14***	7.754*	12.51**	21.11***	8.403*	21.63***
	(4.918)	(3.904)	(3.503)	(3.862)	(3.087)	(4.198)	(3.773)
R-squared	0.276	0.291	0.209	0.194	0.191	0.204	0.183
Adj. R-squared	0.145	0.162	0.065	0.048	0.044	0.060	0.034
Observations	105	105	105	105	105	105	105

OLS coefficients are reported with robust standard errors in parentheses. Each subject is one unit of observation. Two subjects were excluded due to missing responses in the post-experimental questionnaire.

Reference category for treatment is the control treatment (CT), for studies it is Economics, and for gender it is female. Risk attitude and shopping experience were elicited using Likert scales.

The variables Self-reported: ArithmeticMean, Self-reported: Median, Self-reported: FiveStars, Self-reported: OneStar, Self-reported: NegativeReviews, Self-reported: PositiveReviews, and Self-reported: NoPattern are dummy variables derived from the post-experimental questionnaire, indicating whether a subject self-reported following the respective aggregation principle (1) or not (0). Multiple selections were possible.

* *p* < 0.05, ** *p* < 0.01, *** *p* < 0.001

## Conclusion

The aggregation of customer rating distributions into single values is a common practice on online market platforms. These aggregated indicators such as the mean, median, or frequency of extreme ratings are widely regarded as critical signals for consumers in evaluating product quality and making informed purchasing decisions. Despite their central role in digital marketplaces, the underlying cognitive processes through which consumers interpret and process rating distributions remain poorly understood. To address this research gap, we conducted a controlled laboratory experiment in which participants were asked to rank triplets of products characterized by distinct rating distributions according to their personal preferences using an incentive-compatible mechanism. Using the resulting ranking data, we identified consistent patterns in evaluative behavior and systematically compared them against a set of predefined aggregation functions including the arithmetic mean, median, extremity focused weights and binary categorization. This comparative analysis enabled us to assess which aggregation rules best explain observed choice patterns. Based on the category weights estimated through our data model, supported by supplementary robustness checks and model comparisons, we find strong empirical evidence that the majority of consumers aggregate rating distributions in accordance with the arithmetic mean. However, a significant portion of the sample exceeding 40% deviates from this pattern, indicating substantial heterogeneity in evaluative behavior. Our analysis identifies a major cluster of customers who aggregate according to a binary aggregation strategy, assigning equal weight to 1- and 2-star ratings (as negative) and to 4- and 5-star ratings (as positive), thereby collapsing the five-point scale into a dichotomous framework. Other substantial clusters include customers whose decisions closely align with aggregation functions that place primary emphasis on negative feedback, particularly 1-star ratings or the combined frequency of 1- and 2-star ratings.

The robustness of these findings is further reinforced by our result that the identified aggregation patterns remain invariant across conditions in which rating information is presented either as rating distributions accompanied by numerical frequencies and the arithmetic mean or as a purely visual representation. It is important to note that our two treatments were not designed to isolate the effect of any single informational component. Rather, the control treatment, which provides only graphical rating distributions, and the information treatment, which additionally displays numerical frequencies and the arithmetic mean, represent two distinct informational environments at opposite ends of a spectrum. Nevertheless, aggregation behavior remains largely invariant across these two extremes. This indicates that consumers’ preferences for how to process rating information are relatively stable rather than being contingent on the mode of information presentation.

Our study makes several distinct contributions to the literature on consumer decision-making and online reputation systems. First, we provide robust empirical support for the use of the arithmetic mean as a valid and representative aggregation function in the evaluation of product ratings. The strength of this conclusion is further amplified by our methodological design: we employ a controlled laboratory experiment, implement an incentive-compatible ranking mechanism to ensure behavioral authenticity, vary the presentation format of rating information from a purely visual representation to a bundled numerical format, and apply multiple complementary analytical techniques including model estimation and cluster analysis. The consistency of results across these diverse methodological dimensions significantly strengthens the validity and generalizability of our findings.

Second, this study advances beyond prior work that merely investigated whether the arithmetic mean is employed by customers or not. We find that while approximately half of customers apply the arithmetic mean, there is significant heterogeneity in how ratings are aggregated. Our study helps explain this heterogeneity by developing a framework that models product valence using category weights and rating frequencies, allowing us to derive alternative aggregation functions and compare their principles with customers’ ranking decisions. On the one hand, we confirm the practical relevance of the binary aggregation function first experimentally examined by [23]. By demonstrating its use in a more general setting, we show that a substantial group of customers bases their evaluation of product quality on simplified cues, essentially taking the difference between positive (4–5 star) and negative (1–2 star) ratings. This suggests that for many customers, the information in rating distributions is processed through a more cognitively manageable binary categorization. On the other hand, we identify the practical use of aggregation functions that have not been prominently featured in previous research. Prior studies have suggested that customers may be more affected by negative than positive reviews (e.g., [44,45]), an observation theoretically supported by prospect theory [46], which proposes that negative outcomes are weighted more heavily than positive outcomes of equal magnitude. Our study provides direct evidence that this negativity weighting manifests in consumers’ aggregation principles and, consequently, in their judgment of product quality. For some customers, a product’s quality is primarily assessed through the extent to which it has received the least negative experiences. Finally, our findings address the relationship between theoretical elaboration and practical applications of aggregation functions. Although the median has been theoretically evaluated to possess desirable properties for aggregating ratings [4], its practical use appears to be limited. Even more strikingly than previous empirical comparisons between the median and mean [29], we find no evidence that customers use the median as a way to aggregate product ratings. This indicates that aggregation functions may not be actually used even if they are theoretically sound and built on meaningful premises. While this may have been only an exception, research is nevertheless encouraged to put forth an agenda that empirically tests aggregation functions on a systematic basis. Such research could clarify if it is just individual preferences that lead customers to use different aggregation principles than suggested or whether conditions exist that hinder such practices. In this vein, we emphasize our methodological contribution in this research area. We believe that combining a laboratory setting with a data-driven approach offers a promising empirical tool that can complement field studies and qualitative research. For example, a controlled and incentive-compatible setting that reduces the decision environment to the most relevant factors can be a useful first step in identifying and discriminating between aggregation functions, before more context-driven research in the field can be conducted. Similarly, statistical models like the Plackett-Luce model which enable the matching of observed decisions with those obtained from particular reference functions, can better capture the underlying aggregation principles than verbal statements about how one perceived to have aggregated product ratings.

Our research is not without limitations. Although our framework enables the derivation of various aggregation functions based on simple relationships, other decision criteria, such as lexicographic rules, which involve sequential processing, may also be employed [47]. For instance, customers might first select products with 1-star ratings below a specific threshold and then choose the product with the highest share of 5-star ratings from the remaining options. While these sequential patterns are plausible, several arguments suggest that they may not be prevalent. For example, [48] show that simple heuristics are often preferred in decision-making contexts, and simultaneous weight-based aggregation may require less cognitive effort than multi-step sequential processing. Furthermore, the time constraints typical of online shopping environments may discourage elaborate sequential evaluation processes. Additionally, the visual presentation of rating distributions on most platforms displays all rating categories simultaneously, naturally encouraging holistic rather than sequential processing. The literature also suggests that incorporating the time dimension can be beneficial in designing aggregation metrics to mitigate the impact of fraudulent reviews [49] or identify quality changes [50]. While acknowledging these advantages in designing reputation systems, our study focuses on patterns of customers who typically make their purchase decision in a short period, allowing us to suppress the time dimension in our analysis. By abstracting from features of reputation systems that are not relevant to our research question, we strengthen the internal validity of our results.

Our experimental design was deliberately constructed to provide sufficient heterogeneity in rating distributions to identify average ranking behavior and to detect stable patterns of aggregation behavior across subjects. While this approach allowed us to establish the core findings reported above, it also means that several dimensions warrant dedicated investigation in future research. First, with regard to the characteristics of the rating distributions, our product groups were designed to generate enough variation to discriminate between alternative aggregation functions, not to systematically vary specific distributional properties such as dispersion, skewness, or the ranges of mean ratings. Our exploratory analysis at the product level reveals plausible but mainly statistically non-significant relationships between distributional characteristics and departures from the arithmetic mean. A systematic investigation would thus require a dedicated experimental design that independently manipulates these properties across choice sets with a larger number of product groups. Such research could examine not only under which conditions subjects depart from the arithmetic mean, but also under which conditions they are more likely to adopt one of the alternative aggregation functions we identified, such as binary or negative-focused strategies.

Second, with regard to the availability of numerical information, we acknowledge that our information treatment simultaneously introduces numerical frequencies and the explicit arithmetic mean, and therefore does not permit the identification of the effect of each component in isolation. Consequently, we cannot determine whether the graphical representation in the control condition already conveys most of the relevant information, or whether the explicit display of the arithmetic mean guides participants toward a particular aggregation rule. Future research could disentangle these components by varying them independently, for example by including a treatment that provides numerical frequencies without displaying the arithmetic mean. Further, other features such as display formats, rating distributions, product groups and the number of products to be simultaneously ranked may also be explored.

Third, with regard to individual characteristics, our post-experimental questionnaire captured standard demographic and attitudinal variables that could plausibly influence aggregation behavior, including risk preferences, shopping experience, age, and gender. The multivariate analyses reveal that these variables have no substantial impact on aggregation behavior, with the notable exception of a plausible relationship between risk-seeking attitudes and departures from (closer alignment with) negative-focused (5-star-focused) aggregation functions. Importantly, these null results do not imply that individual differences are irrelevant, but rather that the factors we examined are not systematically associated with a tendency towards or against a particular aggregation function. Future research could explore a broader set of individual characteristics, for example by measuring consumers’ sensitivity towards negative experiences, their tendency to simplify cognitively complex tasks, or their propensity to accept information at face value versus critically evaluating it, and investigate whether specific personality traits, attitudes, or reasoning styles predispose consumers toward particular aggregation strategies. Hence, each dimension poses relevant and important research questions in its own right, and dedicated experimental designs that systematically vary distributional properties, measures of individual difference, or informational components could substantially advance our understanding of how consumers process rating information, whether by uncovering fundamental differences in aggregation behavior or by identifying moderators and boundary conditions that clarify the scope of the observed effects.

Despite its limitation, our study carries important practical implications. Given the profound impact of customer ratings on purchase decisions, one of the primary objectives of reputation systems is to ensure that ratings are informative and useful for consumers. Achieving this goal involves two crucial steps. First, platforms must ensure that ratings are provided truthfully, and research has suggested various approaches for how this may be achieved or at least approximated (e.g., incentive mechanisms and/or verification procedures, see for example [51]). The second step, where our research provides guidance, is to present ratings in a way that is helpful and meaningful for customers. Our results suggest that the standard approach of aggregating product ratings using the arithmetic mean may not be sufficiently informative for a substantial portion of customers. Therefore, platform operators may consider offering customers the option to select their preferred aggregation function for product presentation, allowing them to choose a valence calculation procedure that aligns with their personal aggregation principles. Our research has already identified several alternative aggregation functions that customers actually use, such as binary patterns and negative-focused approaches, which could serve as initial options for developing such a customizable system. This approach would have several advantages. First, by customizing the aggregation of product ratings, platform operators would provide a service that acknowledges the heterogeneity of customer preferences, signaling their commitment to assisting customers in making informed purchase decisions. Second, similar to how specialized travel platforms allow singles to focus on reviews from other single travelers rather than reports from couples or families, customized aggregation would present information aligned with individual preferences, potentially saving time and leading to more informed purchase decisions. Furthermore, this tailored approach could lead to increased customer satisfaction, as users would be able to make decisions based on ratings that are more relevant to their needs and preferences. Naturally, implementing different presentation modes and product structuring would require technological development. However, since all the necessary rating data already exists and the task involves calculating valence scores based on different aggregation principles followed by restructuring products according to these calculations, such modifications may be manageable within existing systems. Given that these changes could lead to more satisfied customers who require less time to make decisions, while also attracting new users to the platform, these system investments appear to be highly worthwhile.

## Supporting information

S1 FileS1 Table. Rating distribution of products of different product groups (three products per group).**S2 Table. Category weights of reference functions.** Estimated weights are provided in *-rows. **S3 Table. Distribution of ranking decisions by treatment.** IT = information treatment, CT = control treatment. R1 represents rankings in accordance with the arithmetic mean (1st, 2nd, 3rd), with percentage shares in parentheses. R2 through R6 represent alternative ranking patterns: R2 = (2nd, 1st, 3rd), R3 = (1st, 3rd, 2nd), R4 = (3rd, 1st, 2nd), R5 = (2nd, 3rd, 1st), and R6 = (3rd, 2nd, 1st). See Table S1 for the distribution of each product in each product group. **S4 Table. Characteristics of product groups and AM-consistent rankings.** Mean (A), Mean (B), and Mean (C) denote the arithmetic means of the three products within each product group, ordered from highest to lowest. SD denotes the corresponding standard deviations. Δ Mean and Δ SD capture the difference between the highest and lowest value within each product group. Decision valence is the average of the arithmetic means of the three products within each product group. AM share R1 reports the percentage of subjects whose ranking decision is consistent with the arithmetic mean, separately for the control treatment (CT) and information treatment (IT). **S5 Table. Correlations between product group characteristics and AM-consistent rankings.** Entries report Spearman correlation coefficients (Spearman’s ρ) with p-values in parentheses between the respective characteristic of the product group and the share of AM-consistent rankings across the 12 product groups, separately for the control treatment (CT) and information treatment (IT), and pooled (CT + IT). **S6 Table. Clusters of subjects assigned to reference functions with minimized average Kendall distances (with ties included).** Average and standard deviation (in parentheses) of the estimated model parameters (category weights and precision parameter) are shown. **S7 Table. Clusters of subjects assigned to reference functions with minimized average Kendall distances (with ties excluded).** Average and standard deviation (in parentheses) of the estimated model parameters (category weights and precision parameter) are shown. **S8 Table. Estimated model parameters for participants in the control treatment. S9 Table. Estimated model parameters for participants in the information treatment. S10 Table. Win/tie/loss statistics comparing the estimated model with reference aggregation functions.** Separately for control (top panel) and information (bottom panel) treatment. **S11 Table. Likelihood ratio statistics and corresponding p-values by participant.** In the control treatment (left panel; participant IDs 1–53) and information treatment (right panel; participant IDs 54–107).(ZIP)
