# Supplementary material for: Aggregation processes in customer rating systems - Insights from an economic decision experiment
Source: PLoS One. 2026 Jul 14;21(7):e0343851. doi: 10.1371/journal.pone.0343851 (PMC13367904; doi:10.1371/journal.pone.0343851)
Supplement: S1 File — S2 Table. Category weights of reference functions. Estimated weights are provided in *-rows. S3 Table. Distribution of ranking decisions by treatment. IT = information treatment, CT = control treatment. R1 represents rankings in accordance with the arithmetic mean (1st, 2nd, 3rd), with percentage shares in parentheses. R2 through R6 represent alternative ranking patterns: R2 = (2nd, 1st, 3rd), R3 = (1st, 3rd, 2nd), R4 = (3rd, 1st, 2nd), R5 = (2nd, 3rd, 1st), and R6 = (3rd, 2nd, 1st). See Table S1 for the distribution of each product in each product group. S4 Table. Characteristics of product groups and AM-consistent rankings. Mean (A), Mean (B), and Mean (C) denote the arithmetic means of the three products within each product group, ordered from highest to lowest. SD denotes the corresponding standard deviations. Δ Mean and Δ SD capture the difference between the highest and lowest value within each product group. Decision valence is the average of the arithmetic means of the three products within each product group. AM share R1 reports the percentage of subjects whose ranking decision is consistent with the arithmetic mean, separately for the control treatment (CT) and information treatment (IT). S5 Table. Correlations between product group characteristics and AM-consistent rankings. Entries report Spearman correlation coefficients (Spearman’s ρ) with p-values in parentheses between the respective characteristic of the product group and the share of AM-consistent rankings across the 12 product groups, separately for the control treatment (CT) and information treatment (IT), and pooled (CT + IT). S6 Table. Clusters of subjects assigned to reference functions with minimized average Kendall distances (with ties included). Average and standard deviation (in parentheses) of the estimated model parameters (category weights and precision parameter) are shown. S7 Table. Clusters of subjects assigned to reference functions with minimized average Kendall distances (with tie [file pone.0343851.s001.zip › S11_Table.pdf]

S11 Table. Likelihood ratio statistics and corresponding p-values by participant. In the control treatment (left panel; participant IDs 1-53) and information treatment (right panel; participant IDs 54-107).

ID	z-value	p-value		ID	z-value	p-value
1	14.886	(0.002)		54	5.477	(0.140)
2	5.617	(0.132)		55	2.383	(0.497)
3	20.802	(0.000)		56	14.329	(0.002)
4	1.534	(0.675)		57	3.975	(0.264)
5	11.310	(0.010)		58	15.196	(0.002)
6	7.260	(0.064)		59	14.121	(0.003)
7	4.605	(0.203)		60	15.939	(0.001)
8	0.268	(0.966)		61	13.650	(0.003)
9	3.556	(0.314)		62		
10	14.450	(0.006)		63	0.168	(0.983)
11	13.148	(0.004)		64	12.552	(0.006)
12	15.419	(0.001)		65	6.508	(0.089)
13	9.645	(0.022)		66	10.876	(0.012)
14	9.598	(0.022)		67	2.534	(0.469)
15	13.813	(0.003)		68	3.357	(0.340)
16	6.040	(0.110)		69	9.537	(0.023)
17	1.068	(0.785)		70	14.586	(0.002)
18	3.265	(0.353)		71	3.010	(0.390)
19	15.579	(0.001)		72	4.553	(0.208)
20	17.735	(0.000)		73	18.505	(0.000)
21	1.157	(0.763)		74	4.576	(0.206)
22	1.775	(0.620)		75	18.236	(0.000)
23	0.714	(0.870)		76	4.093	(0.252)
24	1.272	(0.736)		77	13.806	(0.003)
25				78	2.904	(0.407)
26	18.025	(0.000)		79		
27	8.388	(0.039)		80	15.432	(0.001)
28	0.448	(0.930)		81	10.920	(0.012)
29	6.875	(0.076)		82	13.034	(0.005)
30	6.787	(0.079)		83	4.524	(0.210)
31	9.876	(0.020)		84	0.633	(0.889)
32	4.803	(0.187)		85	7.243	(0.065)
33	18.036	(0.000)		86	2.597	(0.458)
34	3.299	(0.348)		87	3.733	(0.292)
35	13.988	(0.003)		88	3.322	(0.345)
36	3.246	(0.355)		89	22.800	(0.000)
37	12.413	(0.006)		90	13.102	(0.004)
38	8.546	(0.036)		91	4.636	(0.200)
39	8.404	(0.038)		92	9.258	(0.026)
40	16.786	(0.001)		93	12.365	(0.006)
41	1.822	(0.610)		94	10.968	(0.012)
42	28.172	(0.000)		95	2.845	(0.416)
43	16.240	(0.001)		96	11.310	(0.010)
44	6.387	(0.094)		97	10.793	(0.013)
45	6.250	(0.100)		98	5.673	(0.129)
46	6.176	(0.103)		99	12.221	(0.007)
47	3.564	(0.313)		100	8.951	(0.030)
48	3.488	(0.322)		101	10.447	(0.015)
49	5.153	(0.161)		102	26.938	(0.000)
50	0.560	(0.906)		103	0.830	(0.842)
51	5.529	(0.137)		104	0.739	(0.864)
52	6.494	(0.090)		105	4.396	(0.222)
53	6.483	(0.090)		106	6.560	(0.087)
				107	1.014	(0.798)

Note: For participants 25, 62, and 79 the log-likelihood value could not be computed (i.e., e(ll) = .), despite successful convergence of the estimation algorithm. This prevented the calculation of likelihood ratio statistics for these cases.

We compute the likelihood ratio statistic Λ between the arithmetic mean (AM) reference function (being the null hypothesis) and the fitted model (maximum likelihood estimate). Since the w_i parameters of the AM reference function are fixed to ($w_1 = -\frac{1}{3}$, $w_2 = -\frac{1}{6}$, $w_3 = 0$, $w_4 = \frac{1}{6}$, $w_5 = \frac{1}{3}$), only the conformity parameter α_i has to be fitted. Therefore, the likelihood ratio statistic is asymptotically χ^2 distributed with 4 degrees of freedom (four independent w_i' parameters in the reparametrized model). The corresponding test statistics and p-values are reported in Table S11. The null-hypothesis is rejected for 48 participants (i.e., 44.86% of participants) at the 5% significance level, providing further support for a proper goodness-of-fit.
