# Supplementary material for: Aggregation processes in customer rating systems - Insights from an economic decision experiment
Source: PLoS One. 2026 Jul 14;21(7):e0343851. doi: 10.1371/journal.pone.0343851 (PMC13367904; doi:10.1371/journal.pone.0343851)
Supplement: S1 File — S2 Table. Category weights of reference functions. Estimated weights are provided in *-rows. S3 Table. Distribution of ranking decisions by treatment. IT = information treatment, CT = control treatment. R1 represents rankings in accordance with the arithmetic mean (1st, 2nd, 3rd), with percentage shares in parentheses. R2 through R6 represent alternative ranking patterns: R2 = (2nd, 1st, 3rd), R3 = (1st, 3rd, 2nd), R4 = (3rd, 1st, 2nd), R5 = (2nd, 3rd, 1st), and R6 = (3rd, 2nd, 1st). See Table S1 for the distribution of each product in each product group. S4 Table. Characteristics of product groups and AM-consistent rankings. Mean (A), Mean (B), and Mean (C) denote the arithmetic means of the three products within each product group, ordered from highest to lowest. SD denotes the corresponding standard deviations. Δ Mean and Δ SD capture the difference between the highest and lowest value within each product group. Decision valence is the average of the arithmetic means of the three products within each product group. AM share R1 reports the percentage of subjects whose ranking decision is consistent with the arithmetic mean, separately for the control treatment (CT) and information treatment (IT). S5 Table. Correlations between product group characteristics and AM-consistent rankings. Entries report Spearman correlation coefficients (Spearman’s ρ) with p-values in parentheses between the respective characteristic of the product group and the share of AM-consistent rankings across the 12 product groups, separately for the control treatment (CT) and information treatment (IT), and pooled (CT + IT). S6 Table. Clusters of subjects assigned to reference functions with minimized average Kendall distances (with ties included). Average and standard deviation (in parentheses) of the estimated model parameters (category weights and precision parameter) are shown. S7 Table. Clusters of subjects assigned to reference functions with minimized average Kendall distances (with tie [file pone.0343851.s001.zip › S1_Table.pdf]

S1 Table. Rating distribution of products of different product groups (three products per group).

product_i	$x_{i,1}$	$x_{i,2}$	$x_{i,3}$	$x_{i,4}$	$x_{i,5}$	Mean value	Median
1	0.086	0.028	0.044	0.126	0.716	4.358	5
2	0.074	0.025	0.074	0.222	0.605	4.259	5
3	0.069	0.080	0.103	0.149	0.599	4.129	5
4	0.038	0.055	0.066	0.159	0.682	4.392	5
5	0.027	0.054	0.081	0.257	0.581	4.311	5
6	0.102	0.082	0.020	0.061	0.735	4.245	5
7	0.154	0.077	0.115	0.038	0.616	3.885	5
8	0.149	0.085	0.064	0.213	0.489	3.808	4
9	0.162	0.030	0.131	0.273	0.404	3.727	4
10	0.140	0.065	0.061	0.170	0.564	3.953	5
11	0.021	0.191	0.120	0.291	0.377	3.812	4
12	0.061	0.087	0.122	0.700	0.030	3.551	4
13	0.017	0.089	0.400	0.106	0.388	3.759	3
14	0.056	0.133	0.193	0.346	0.272	3.645	4
15	0.240	0.111	0.135	0.005	0.509	3.432	5
16	0.000	0.048	0.504	0.087	0.361	3.761	3
17	0.195	0.012	0.145	0.259	0.389	3.635	4
18	0.246	0.150	0.091	0.012	0.501	3.372	5
19	0.017	0.276	0.208	0.020	0.479	3.668	3
20	0.175	0.058	0.149	0.293	0.325	3.535	4
21	0.335	0.012	0.134	0.007	0.512	3.349	5
22	0.034	0.211	0.256	0.076	0.423	3.643	3
23	0.249	0.052	0.017	0.367	0.315	3.447	4
24	0.411	0.012	0.074	0.000	0.503	3.172	5
25	0.290	0.216	0.000	0.129	0.365	3.063	2
26	0.262	0.030	0.402	0.164	0.142	2.894	3
27	0.447	0.000	0.048	0.384	0.121	2.732	4
28	0.165	0.074	0.057	0.102	0.602	3.902	5
29	0.255	0.053	0.051	0.119	0.522	3.600	5
30	0.214	0.071	0.143	0.143	0.429	3.502	4
31	0.059	0.029	0.029	0.147	0.736	4.472	5
32	0.097	0.065	0.000	0.129	0.709	4.288	5
33	0.143	0.024	0.000	0.119	0.714	4.237	5
34	0.039	0.039	0.066	0.158	0.698	4.437	5
35	0.097	0.065	0.065	0.065	0.708	4.222	5
36	0.115	0.082	0.033	0.180	0.590	4.048	5
