# Supplementary material for: Aggregation processes in customer rating systems - Insights from an economic decision experiment
Source: PLoS One. 2026 Jul 14;21(7):e0343851. doi: 10.1371/journal.pone.0343851 (PMC13367904; doi:10.1371/journal.pone.0343851)
Supplement: S1 File — S2 Table. Category weights of reference functions. Estimated weights are provided in *-rows. S3 Table. Distribution of ranking decisions by treatment. IT = information treatment, CT = control treatment. R1 represents rankings in accordance with the arithmetic mean (1st, 2nd, 3rd), with percentage shares in parentheses. R2 through R6 represent alternative ranking patterns: R2 = (2nd, 1st, 3rd), R3 = (1st, 3rd, 2nd), R4 = (3rd, 1st, 2nd), R5 = (2nd, 3rd, 1st), and R6 = (3rd, 2nd, 1st). See Table S1 for the distribution of each product in each product group. S4 Table. Characteristics of product groups and AM-consistent rankings. Mean (A), Mean (B), and Mean (C) denote the arithmetic means of the three products within each product group, ordered from highest to lowest. SD denotes the corresponding standard deviations. Δ Mean and Δ SD capture the difference between the highest and lowest value within each product group. Decision valence is the average of the arithmetic means of the three products within each product group. AM share R1 reports the percentage of subjects whose ranking decision is consistent with the arithmetic mean, separately for the control treatment (CT) and information treatment (IT). S5 Table. Correlations between product group characteristics and AM-consistent rankings. Entries report Spearman correlation coefficients (Spearman’s ρ) with p-values in parentheses between the respective characteristic of the product group and the share of AM-consistent rankings across the 12 product groups, separately for the control treatment (CT) and information treatment (IT), and pooled (CT + IT). S6 Table. Clusters of subjects assigned to reference functions with minimized average Kendall distances (with ties included). Average and standard deviation (in parentheses) of the estimated model parameters (category weights and precision parameter) are shown. S7 Table. Clusters of subjects assigned to reference functions with minimized average Kendall distances (with tie [file pone.0343851.s001.zip › S8_Table.pdf]

S8 Table. Estimated model parameters for participants in the control treatment.

ID	w1	w2	w3	w4	w5	alpha
1	-0.237	-0.061	-0.202	0.132	0.368	58.2
2	-0.237	-0.263	0.002	0.185	0.314	69.1
3	-0.191	-0.231	-0.078	0.026	0.474	58.9
4	-0.304	-0.005	-0.191	0.115	0.385	12.5
5	-0.401	-0.072	-0.027	0.258	0.242	59.5
6	-0.172	-0.221	-0.107	0.229	0.271	42.6
7	-0.353	0.102	-0.147	0.208	0.190	24.3
8	-0.323	-0.177	0.021	0.166	0.313	62.7
9	-0.310	-0.190	0.004	0.247	0.248	30.1
10	-0.370	-0.130	0.178	0.053	0.269	3570.9
11	-0.321	-0.122	-0.057	0.220	0.280	236.1
12	-0.448	-0.052	0.063	0.271	0.166	55.4
13	-0.292	-0.208	0.005	0.240	0.255	79.3
14	-0.379	-0.089	-0.032	0.253	0.247	78.4
15	-0.272	-0.216	-0.013	0.207	0.293	328.7
16	-0.293	-0.056	-0.151	0.234	0.266	35.6
17	-0.322	-0.178	0.006	0.195	0.299	54.9
18	-0.415	-0.058	-0.027	0.200	0.300	62.0
19	-0.196	-0.239	-0.065	0.200	0.300	113.6
20	-0.338	-0.162	0.057	0.199	0.244	1308.9
21	-0.299	-0.190	-0.011	0.197	0.303	46.0
22	-0.271	-0.160	-0.070	0.296	0.204	13.9
23	-0.351	-0.126	-0.023	0.191	0.309	47.8
24	-0.340	-0.160	0.003	0.201	0.296	58.7
25	-0.500	0.151	0.032	0.119	0.198	4875.0
26	-0.324	-0.176	0.034	0.213	0.253	491.8
27	-0.403	0.057	-0.097	0.277	0.166	34.5
28	-0.318	-0.153	-0.028	0.177	0.323	56.0
29	-0.300	-0.200	0.282	0.029	0.190	41.5
30	-0.267	-0.233	0.276	0.124	0.099	24.2
31	0.008	0.079	-0.382	0.413	-0.118	14.8
32	-0.256	-0.244	0.024	0.216	0.260	45.3
33	-0.292	-0.139	-0.069	0.234	0.266	148.0
34	-0.493	0.273	-0.007	0.071	0.155	14.0
35	-0.312	-0.182	-0.005	0.236	0.264	149.1
36	-0.267	-0.204	-0.029	0.153	0.347	100.8
37	-0.302	-0.198	0.038	0.208	0.254	183.9
38	-0.280	-0.220	0.034	0.213	0.254	76.8
39	-0.290	-0.208	-0.002	0.239	0.261	74.1
40	-0.437	-0.063	0.010	0.243	0.247	172.8
41	-0.385	-0.095	-0.020	0.208	0.292	43.7
42	-0.347	-0.153	0.040	0.242	0.218	358.2
43	-0.002	0.500	-0.062	-0.010	-0.427	38.3
44	-0.353	-0.099	-0.048	0.253	0.247	42.5
45	-0.325	-0.162	-0.014	0.220	0.280	148.5
46	-0.355	-0.145	0.162	0.113	0.225	55.7
47	-0.253	-0.112	-0.134	0.224	0.276	34.5
48	-0.413	0.004	-0.087	0.117	0.379	74.1
49	-0.339	-0.120	-0.041	0.232	0.268	56.4
50	-0.327	-0.173	0.022	0.140	0.338	64.9
51	-0.254	-0.246	0.006	0.108	0.386	54.3
52	-0.253	-0.247	0.041	0.108	0.351	82.0
53	-0.486	-0.014	0.092	0.156	0.252	48.1
