# Supplementary material for: Aggregation processes in customer rating systems - Insights from an economic decision experiment
Source: PLoS One. 2026 Jul 14;21(7):e0343851. doi: 10.1371/journal.pone.0343851 (PMC13367904; doi:10.1371/journal.pone.0343851)
Supplement: S1 File — S2 Table. Category weights of reference functions. Estimated weights are provided in *-rows. S3 Table. Distribution of ranking decisions by treatment. IT = information treatment, CT = control treatment. R1 represents rankings in accordance with the arithmetic mean (1st, 2nd, 3rd), with percentage shares in parentheses. R2 through R6 represent alternative ranking patterns: R2 = (2nd, 1st, 3rd), R3 = (1st, 3rd, 2nd), R4 = (3rd, 1st, 2nd), R5 = (2nd, 3rd, 1st), and R6 = (3rd, 2nd, 1st). See Table S1 for the distribution of each product in each product group. S4 Table. Characteristics of product groups and AM-consistent rankings. Mean (A), Mean (B), and Mean (C) denote the arithmetic means of the three products within each product group, ordered from highest to lowest. SD denotes the corresponding standard deviations. Δ Mean and Δ SD capture the difference between the highest and lowest value within each product group. Decision valence is the average of the arithmetic means of the three products within each product group. AM share R1 reports the percentage of subjects whose ranking decision is consistent with the arithmetic mean, separately for the control treatment (CT) and information treatment (IT). S5 Table. Correlations between product group characteristics and AM-consistent rankings. Entries report Spearman correlation coefficients (Spearman’s ρ) with p-values in parentheses between the respective characteristic of the product group and the share of AM-consistent rankings across the 12 product groups, separately for the control treatment (CT) and information treatment (IT), and pooled (CT + IT). S6 Table. Clusters of subjects assigned to reference functions with minimized average Kendall distances (with ties included). Average and standard deviation (in parentheses) of the estimated model parameters (category weights and precision parameter) are shown. S7 Table. Clusters of subjects assigned to reference functions with minimized average Kendall distances (with tie [file pone.0343851.s001.zip › S9_Table.pdf]

S9 Table. Estimated model parameters for participants in the information treatment.

ID	w1	w2	w3	w4	w5	alpha
54	-0.270	-0.180	-0.050	0.167	0.333	123.7
55	-0.414	-0.086	0.029	0.399	0.072	6.6
56	-0.474	0.134	0.150	0.216	-0.026	28.4
57	-0.263	-0.234	-0.003	0.176	0.324	108.7
58	-0.304	-0.196	0.052	0.216	0.232	138.8
59	-0.296	-0.204	0.073	0.183	0.243	356.6
60	-0.263	0.357	0.143	-0.137	-0.100	52.4
61	-0.418	0.246	-0.040	0.254	-0.042	33.1
62	-0.293	-0.207	0.012	0.226	0.263	3483.3
63	-0.322	-0.178	0.007	0.173	0.321	101.7
64	-0.260	-0.178	-0.062	0.252	0.248	71.7
65	-0.188	-0.193	0.500	-0.090	-0.030	16.1
66	-0.306	-0.194	0.029	0.209	0.262	208.3
67	-0.307	-0.193	0.119	0.136	0.244	34.4
68	-0.361	-0.139	0.026	0.212	0.262	39.7
69	-0.261	-0.201	-0.038	0.205	0.295	114.3
70	-0.283	-0.217	0.061	0.276	0.163	50.6
71	-0.333	-0.141	-0.027	0.100	0.400	34.9
72	-0.317	-0.153	-0.029	0.223	0.277	65.7
73	-0.260	-0.240	0.005	0.216	0.278	447.3
74	-0.309	-0.191	0.006	0.222	0.271	70.4
75	-0.290	-0.210	0.016	0.224	0.260	271.4
76	-0.277	-0.211	-0.012	0.247	0.253	32.7
77	-0.315	-0.183	-0.002	0.222	0.278	528.0
78	-0.334	-0.083	-0.083	0.194	0.306	54.5
79	-0.290	-0.186	-0.024	0.205	0.295	3163.9
80	-0.291	-0.194	-0.014	0.226	0.274	245.3
81	-0.304	-0.196	0.068	0.206	0.226	77.3
82	-0.231	-0.269	0.015	0.165	0.320	191.8
83	-0.267	-0.233	0.057	0.182	0.261	57.3
84	-0.384	0.084	-0.116	0.174	0.243	10.4
85	-0.313	-0.157	-0.029	0.214	0.286	214.3
86	-0.500	0.016	0.364	0.102	0.019	6.6
87	-0.439	0.080	-0.061	0.049	0.371	23.4
88	-0.380	-0.120	0.025	0.094	0.381	47.2
89	-0.301	-0.199	0.021	0.234	0.245	319.1
90	-0.321	-0.178	-0.001	0.225	0.275	409.8
91	-0.371	-0.024	-0.105	0.081	0.419	33.2
92	-0.266	-0.226	-0.008	0.216	0.284	108.7
93	-0.249	-0.251	0.055	0.245	0.199	52.1
94	-0.338	-0.119	-0.043	0.231	0.269	134.4
95	-0.336	-0.142	-0.022	0.216	0.284	66.7
96	-0.227	-0.218	-0.055	0.218	0.282	87.2
97	-0.326	-0.174	0.056	0.224	0.220	59.3
98	-0.362	0.037	-0.138	0.126	0.337	50.7
99	-0.355	-0.030	-0.115	0.136	0.364	198.5
100	-0.381	-0.034	-0.085	0.290	0.210	41.2
101	-0.241	-0.259	0.008	0.228	0.264	68.9
102	-0.265	-0.235	0.191	0.132	0.176	370.8
103	-0.320	-0.170	-0.010	0.201	0.299	43.8
104	-0.293	-0.207	0.022	0.153	0.325	91.5
105	-0.205	0.486	-0.040	0.014	-0.254	12.1
106	-0.356	-0.144	0.006	0.232	0.262	87.3
107	0.298	0.121	0.082	-0.201	-0.299	28.3
